# Empagliflozin mitigates type 2 diabetes-associated peripheral neuropathy: a glucose-independent effect through AMPK signaling

**DOI:** 10.1007/s12272-022-01391-5

**Published:** 2022-06-29

**Authors:** Noha F. Abdelkader, Marawan A. Elbaset, Passant E. Moustafa, Sherehan M. Ibrahim

**Affiliations:** 1 Department of Pharmacology and Toxicology, Faculty of Pharmacy, Cairo University, Kasr El-Aini St., Cairo, 11562 Egypt; 2 Medical Research and Clinical Studies Institute, Pharmacology, National Research Centre, Giza, Egypt

**Keywords:** Empagliflozin, Dorsomorphin, Diabetic peripheral neuropathy, AMPK, p38 MAPK, mTOR

## Abstract

Diabetic peripheral neuropathy (DPN) represents a severe microvascular condition that dramatically affects diabetic patients despite adequate glycemic control, resulting in high morbidity. Thus, recently, anti-diabetic drugs that possess glucose-independent mechanisms attracted attention. This work aims to explore the potentiality of the selective sodium-glucose cotransporter-2 inhibitor, empagliflozin (EMPA), to ameliorate streptozotocin-induced DPN in rats with insight into its precise signaling mechanism. Rats were allocated into four groups, where control animals received vehicle daily for 2 weeks. In the remaining groups, DPN was elicited by single intraperitoneal injections of freshly prepared streptozotocin and nicotinamide (52.5 and 50 mg/kg, respectively). Then EMPA (3 mg/kg/p.o.) was given to two groups either alone or accompanied with the AMPK inhibitor dorsomorphin (0.2 mg/kg/i.p.). Despite the non-significant anti-hyperglycemic effect, EMPA improved sciatic nerve histopathological alterations, scoring, myelination, nerve fibers’ count, and nerve conduction velocity. Moreover, EMPA alleviated responses to different nociceptive stimuli along with improved motor coordination. EMPA modulated ATP/AMP ratio, upregulated p-AMPK while reducing p-p38 MAPK expression, p-ERK1/2 and consequently p-NF-κB p65 as well as its downstream mediators (TNF-α and IL-1β), besides enhancing SOD activity and lowering MDA content. Moreover, EMPA downregulated mTOR and stimulated ULK1 as well as beclin-1. Likewise, EMPA reduced miR-21 that enhanced RECK, reducing MMP-2 and -9 contents. EMPA’s beneficial effects were almost abolished by dorsomorphin administration. In conclusion, EMPA displayed a protective effect against DPN independently from its anti-hyperglycemic effect, probably via modulating the AMPK pathway to modulate oxidative and inflammatory burden, extracellular matrix remodeling, and autophagy.

**Supplementary Information:**

The online version contains supplementary material available at 10.1007/s12272-022-01391-5.

## Introduction

Diabetes Mellitus (DM) is an endocrine, metabolic disease characterized by either insulin resistance or partial/complete deficiency in pancreatic insulin secretion, resulting in persistently elevated blood glucose levels (American Diabetes Association 2009). DM incidence and prevalence are widely growing to become a pandemic by 2030, as expected by the International Diabetes Federation (Saeedi et al. 2019). Diabetic neuropathy is among the utmost serious diabetic microvascular complications, which affects about half of the patients with DM type 1 and 2 (Lederman 2012), targeting the peripheral nervous system’s sensory, motor, and autonomic neurons (Duby et al. 2004).

Diabetic peripheral neuropathy (DPN), the utmost frequent type of diabetic neuropathy, can lead to foot ulceration with an increased risk of lower limb amputation (Khdour 2020). Patients suffer from numbness and hot sensations (Backonja and Stacey 2004), besides sensory pain affecting distal limbs (Dworkin et al. 2007). The associated neuropathic pain varies in its severity, resulting in a negative impact on patients’ life as well as burdening them with high health care costs (Sadosky et al. 2015). Furthermore, nerve motor dysfunction may trigger muscle weakness and uncontrolled balance (Khdour 2020).

Multiple established mechanisms have been implicated in DPN pathophysiology viz. protein kinase C, the polyol pathways, the formation of the advanced glycation end products, and oxidative stress (Duby et al. 2004). Focusing on oxidative stress caused by hyperglycemia (Obrosova 2002; Vincent et al. 2004), nerve damage could be mediated via inducing nerve microangiopathy and vascular abnormalities (Cameron et al. 2001). These abnormalities are strongly connected to reactive oxygen species (ROS) production, lipid peroxidation, along with a drop in body antioxidant defense mechanisms such as scavenger molecules (Obrosova 2002). Subsequentially, nerve energy production is reduced (Obrosova 2002; Vincent et al. 2004), accompanied by a disturbance in proteins axonal transport (Fernyhough and Schmidt 2002). In crosstalk between hyperglycemia, mitochondrial dysfunction, and oxidative stress, elevated intracellular glucose concentration stimulated mitochondrial NADH, thus electron availability to the respiratory chain, resulting in ROS production (Nishikawa et al. 2000a, b).

Adenosine monophosphate activated protein kinase (AMPK) is a possible target molecule for treating DPN by maintaining cellular energy balance by increasing adenosine triphosphate (ATP)-generating catabolic processes and reducing ATP consuming-anabolic processes (Shrikanth and Nandini 2020). Moreover, Roy Chowdhury et al. (2012) revealed that diminished AMPK cascade interfered with mitochondrial dysfunction and neuronal damage. On the contrary, AMPK pathway activation prevented streptozotocin (STZ)-induced neuroinflammation in experimental animals via stimulating mitochondrial biogenesis and autophagy (Yerra and Kumar 2017). Likewise, it enhanced the expression of antioxidant enzymes in an in vitro experiment (Lin et al. 2017).

Though adequate glycemic control, particularly in type 2 diabetic patients, can still develop DPN among several microvascular complications. Hence, in recent years, much attention has been directed toward new anti-diabetic drugs that can mitigate DPN based on glucose-independent mechanisms (Lee et al. 2018; Eid et al. 2020). Empagliflozin (EMPA) is a selective sodium-glucose cotransporter-2 (SGLT-2) inhibitor, hence, impedes glucose reabsorption from the kidney’s proximal tubules (Scott 2014), resulting in reduced blood glucose level and eventually controlling DM type 2 (Grempler et al. 2012). Regardless of EMPA anti-hyperglycemic effect, it showed a promising efficacy against cardiovascular complications in DM type 2 patients (Zelniker et al. 2019) in addition to heart failure, among other cardiovascular diseases (Zhou and Wu 2017; Packer et al. 2017). Such effect could be attributed to the interaction between renal SGLT-2 and sodium-hydrogen exchange which displays high activity in heart failure, contributing to insensitivity to either diuretic treatments or endogenous natriuretic peptides (Scott 2014). Likewise, EMPA inhibited cardiac sodium-hydrogen exchange, thus reducing cardiac damage, hypertrophy, and remodeling (Scott 2014). Additionally, EMPA’s beneficial effects could be mediated by lowering fluid retention, body weight, blood pressure, renal inflammation, and oxidative stress (Scott 2014; Lee et al. 2018). Accordingly, EMPA represented an excellent candidate to evaluate its possible glucose-dependent and -independent protective effects against diabetic nephropathy, as shown in several investigations (Gembardt et al. 2014; Elrouby and Toural 2017; Eid et al. 2020).

Few recent studies have emphasized the possible role of EMPA in competing against peripheral neuropathy. Lee et al. (2018) revealed that EMPA ameliorated DPN in the DM type 1 rat model in their preliminary research. Likewise, Eid et al. (2020) reported a similar effect in the DM type 1 *db*/+ mouse model; however, EMPA unexpectedly didn’t improve DPN in the DM type 2 *db/db* mouse model. Since SGLT-2 inhibitors are currently not approved for DM type 1, further investigations using different DM type 2 animal models are warranted to verify EMPA efficiency against DPN (Lee et al. 2018; Eid et al. 2020). Furthermore, the possible signaling pathways underlying the glucose-independent protective effect of EMPA have yet to be determined. Hence, the goal of this study was to elucidate the therapeutic impact of EMPA to ameliorate DPN in STZ-induced DM type 2 in rats. Besides, to explore EMPA’s possible mechanistic pathway targeting AMPK and corresponding downstream mediators that may intersect with each other using the AMPK inhibitor dorsomorphin (DORS).

## Materials and methods

### Animals

Adult male Wistar rats (170–220 g) have been acquired from the National Research Center (Giza, Egypt). In the animal house of the Faculty of Pharmacy, Cairo University (Cairo, Egypt), animals were kept for a week to adapt prior to experiments conduction. They were housed under controlled, standardized conditions with a temperature of 22 ± 2 °C, relative humidity of 60 ± 10%, and a 12 h light/dark cycle. All rats had unlimited access to tap water and standard laboratory chow. The Ethics Committee of Cairo University, Faculty of Pharmacy approved this study (Number: PT 1393) which followed the US National Institutes of Health’s Guide for the Care and Use of Laboratory Animals (NIH Publication No. 85-23, revised 2011).

### Drugs and chemicals

Empagliflozin was acquired from Boehringer Ingelheim Pharmaceuticals (Ingelheim, Germany), while DORS and STZ were supplied by Sigma-Aldrich Co. (St. Louis, MO, USA). Nicotinamide (NA) was provided by Bayer (Lyon, France). Every other chemical utilized during experiments was of analytical grade and maximum purity. Freshly suspended EMPA in 1% tween 80 solution was orally administered at a dosage of 3 mg/kg/day (Lee et al. 2018), while DORS was freshly dissolved in 1% dimethyl sulfoxide (DMSO) and intraperitoneally injected at a dosage of 0.2 mg/kg/day (Hasanvand et al. 2018). In addition, NA (50 mg/kg) and STZ (52.5 mg/kg) were freshly solubilized before use in normal physiological saline and citrate buffer (0.1 M, pH 4.5), respectively (Moustafa et al. 2018a; Abdelkader et al. 2022).

### Induction of diabetes

Nicotinamide was intraperitoneally given to overnight-fasted rats 15 min prior to STZ intraperitoneal injection (Moustafa et al. 2018a; Abdelkader et al. 2022). Administration of NA preceded STZ to preserve insulin-secreting β-cells from the damaging effect of STZ partly (Supplementary Fig. 1). Following STZ administration, all rats were given a glucose solution (5%) rather than tap water for 24 h to avoid death from hypoglycemic shock. After that, blood samples were collected from the rat’s tail vein to assess blood glucose levels (BGL) using an ACCU-Check portable glucometer (Roche, Indianapolis, IN, USA) 2 days after STZ administration. Only rats with BGL levels ≥ 200 mg/dl were chosen as diabetic rats (Moustafa et al. 2018b; Mohamed et al. 2020).

### Experimental design

**Fig. 1:**
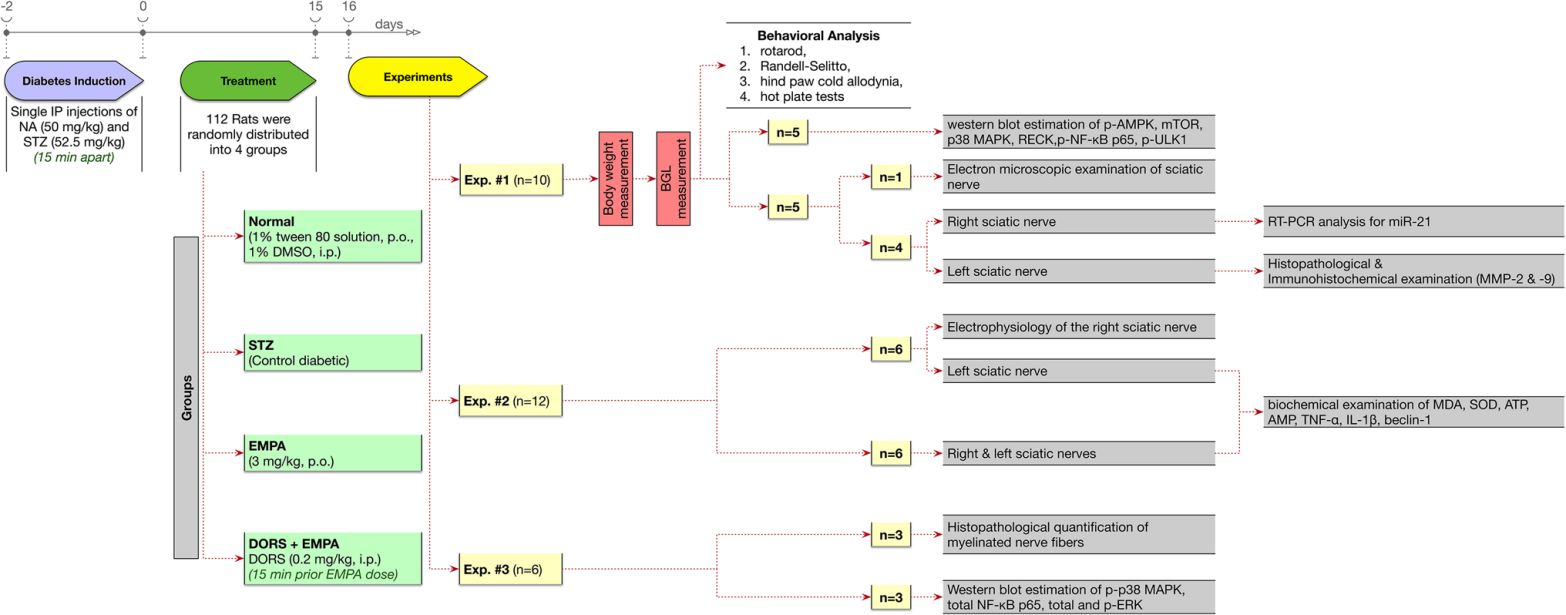
Experimental design

As shown in Fig. 1, the current study consisted of 3 independent experiments (112 rats). In the first experiment, 40 rats were randomly allocated into 4 groups (10 rats/group). In group I, rats received 1% tween 80 solution orally and 1% DMSO intraperitoneally for 15 days and acted as a control. Group II included STZ-induced diabetic rats and served as a diabetic group (STZ group). Diabetic rats in group III were given EMPA (3 mg/kg, p.o.) every day for 15 days. The dose of EMPA was selected from the previous study of Lee et al. (2018). Diabetic rats in group IV were given EMPA (3 mg/ kg, p.o.) and DORS (0.2 mg/ kg, i.p.) daily for 15 days.

All rats were exposed to behavioral analysis 1 day after receiving the last drugs, ordered from the least to the most stressful test: rotarod, Randell-Selitto, hind paw cold allodynia, and finally hot plate. These tests were performed in a sound-isolated room during the light phase, with a 1-h break between the experiments (Abdelkader et al. 2017). After that, rats were weighed, and blood samples were collected from the rat’s tail vein to assess BGL using the portable glucometer. Subsequently, rats were split randomly into 2 sets and were euthanized by decapitation. In the first set (n = 5), both sciatic nerves have been quickly separated, rinsed by ice-cold saline, and then homogenized in a lysis buffer containing a complete protease inhibitor complex. The homogenates were separated by centrifugation at 15,000×*g* for 15 min at 4 °C. The supernatants were divided into aliquots then stored at − 80 °C for later western blot determination of phosphorylated AMPK, mammalian target of rapamycin (mTOR), mitogen-activated protein kinase (p38 MAPK), reversion-inducing cysteine-rich protein with Kazal motifs (RECK), phosphorylated nuclear factor kappa-B (NF-κB) p65, and phosphorylated Unc-51 like autophagy activating kinase 1 (ULK1). In the second set (n = 5), sciatic nerves from one rat/group were fixed overnight in glutaraldehyde (2.5%) in cacodylate buffer (0.1 M, pH 7.4) for electron microscopic examination. Regarding the remaining 4 rats/group, each rat’s right sciatic nerve was promptly extracted, washed, and frozen in liquid nitrogen before storing at − 80 °C till their use to determine microRNA-21 (miR-21) by quantitative real-time PCR. Also, the left sciatic nerves were fixed overnight in neutral-buffered formalin (10%) for histopathological examination and immunohistochemical determination of matrix metalloproteinase (MMP)-2 and MMP-9.

In the second experiment, 48 rats were randomized between 4 groups (12 rats/group) using the same design implemented in the first experiment. One day after receiving the last drugs, electrophysiology analysis for the right sciatic nerves of 6 rats from each group was conducted. After that, all the rats were decapitated. The sciatic nerve tissues on both sides were cautiously excised (excluding the right sciatic nerves used in the electrophysiology experiment), cleaned with ice-cold saline, dried, and weighted. The sciatic nerves from every 2 rats were pooled and homogenized in phosphate buffer to estimate the biochemical parameters (n = 6); malondialdehyde (MDA), superoxide dismutase (SOD), ATP, adenosine monophosphate (AMP), tumor necrosis factor (TNF)-α, interleukin 1β (IL-1β), mammalian orthologue of yeast Atg6 (beclin-1).

In the third experiment, 24 rats were randomized between 4 groups (6 rats/group) using the same design implemented in the previous experiments. One day after receiving the last drugs, rats were split randomly into 2 sets and were decapitated, then the sciatic nerves were carefully excised and washed with ice-cold saline. The sciatic nerves from the first set (n = 3) were fixed overnight in neutral-buffered formalin (10%) to quantify myelinated nerve fibers. In the second set (n = 3), sciatic nerves were processed as previously described for western blot estimation of phosphorylated p38 MAPK and total NF-κB p65 as well as total and phosphorylated extracellular-signal-regulated kinase (ERK). To avoid experimental bias, blinding of all the samples was applied during analysis.

### Body weight change

Each animal’s body weight was measured on the first and last days of the experiment, and the percent change in body weight was calculated using the equation below:

$$\% {\text{Change in body weight}} = \frac{{{\text{body weight in last day}} - {\text{body weight in first day}}}}{{{\text{body weight in first day}}}} \times 100$$

### Behavioral analysis

#### Rotarod test

The motor coordination and balance of rats were evaluated using the Ugo Basile accelerated rotarod apparatus (Model 47750, Italy), where rats were placed in the opposite direction of the rotating rod at a starting speed of 4, which was linearly increased to 40 rpm. Before the experiment, all rats were trained for three consecutive days (one session per day, 5 min each). Each rat’s performance was assessed by recording its time to fall off the rod during a 5-min trial (Lundblad et al. 2003).

#### Randell-Selitto test

Mechanical hypersensitivity was evaluated by the Ugo Basile analgesimeter apparatus (Model 7200, Italy). The dorsal surface of the rat’s left hind paw was pressed by constantly rising pressure till vocalization or paw withdrawal reflex occurred. Rats were gently confined with a soft cloth to immobilize them while measuring the mechanical withdrawal threshold. A cut-off pressure force of 250 g was adopted to prevent tissue injury (Leighton et al. 1988).

#### Hind paw cold allodynia test

The cold pain sensitivity of rats was assessed by gently submerging each rat’s hind paws in an ice-cold water tank, maintained at a constant temperature of 4 ± 1 °C. The hind paw withdrawal latency was measured for each rat. The test was performed twice for every hind paw at a 5-min interval to every rat, and the withdrawal latency was calculated as the mean of both hind paw’s results. Only one hind paw was measured during each immersion, with a cut-off time of 20 s, to avoid tissue damage. A shorter contact time with ice-cold water can be perceived as very severe allodynia (Ameyaw et al. 2014).

#### Hot Plate test

Rats’ heat pain sensitivity was evaluated using the Ugo Basile hot plate apparatus (Model 7280, Italy). Individual rats have been placed on the heated plate fixed at a temperature of 55 ± 1 °C, and latency to withdraw or lick the hind paws or jump to avoid heat pain was recorded as hot plate reaction latency; with a cut-off time of 20 s (Kamel et al. 2022).

### Electrophysiology of sciatic nerve

The nerve conduction velocities, sensory (SNCV) and motor (MNCV), have been recorded as described earlier (Ling et al. 2019; Fontanesi et al. 2019). Rats were anesthetized using a solution of xylazine and ketamine (20 and 50 mg/kg, i.p., respectively). The right sciatic nerve was stimulated using the Ugo Basil ECT Unit (Model 57800, Italy) with the following settings: duration of 0.1 ms, intensity of 20 μA, and frequency of 50 Hz. Then the action potential was recorded by PowerLab 8SP (AD Instruments, Australia) at 10 Hz. The distance between distal and proximal cathodes was divided by the latency difference between proximal and distal cathodes to determine SNCV and MNCV.

### Biochemical analysis

#### Western blot analysis

Protein contents were quantified in sciatic supernatants by a protein assay kit (Bio-Rad, Hercules, CA, USA). Then, protein samples were isolated onto a nitrocellulose membrane using sodium dodecyl sulfate–polyacrylamide gel electrophoresis (Amersham Bioscience, Piscataway, NJ, USA). Membranes were blocked with a 5% non-fat dry milk solution in Tris-buffered saline with Tween (TBST) for 1 h at room temperature. After that, membranes were incubated overnight at 4 °C with 1:1000 dilutions of the primary antibodies: rabbit polyclonal anti-mTOR (Catalog No: ab2732), mouse polyclonal anti-RECK (Catalog No: ab88249), rabbit polyclonal anti-p(Ser536)-NF-κB p65 (Catalog No: ab28856) from Abcam (Waltham, MA, USA), besides rabbit polyclonal anti-p(Ser317)-ULK1 (Catalog No: 37762), rabbit polyclonal anti-p(Thr172)-AMPK (Catalog No: 2535), rabbit monoclonal anti-p(Thr180/Tyr182)-p38 MAPK (Catalog No: 4511), and rabbit monoclonal anti-NF-κB p65 (Catalog No:8242) from Cell Signaling Technology (Danvers, MA, USA), in addition to rabbit polyclonal anti-p38 MAPK (Catalog No: AHO1202), rabbit polyclonal anti-ERK1/2 (Catalog No: 61-7400), rabbit polyclonal anti-p(Thr202/Tyr204)-ERK1/2 (Catalog No: 36-8800) from Thermo Fisher Scientific (Hanover, IL, USA). Following washing with TBST, horseradish peroxidase-conjugated goat anti-mouse immunoglobulin was used to probe the membranes (Life Science Inc., Chicago, IL, USA). Finally, following the manufacturer’s procedures, protein bands were visualized using an enhanced chemiluminescence kit (Amersham Bioscience, Piscataway, NJ, USA). Densitometric analysis of the protein bands was performed using a scanning laser densitometer (Biomed Instrument Inc., Brooklyn, NY, USA), and results were normalized to β-actin protein expression.

#### Quantitative real-time RT-PCR analysis

Total RNAs were isolated from all samples by miRNeasy Serum/Plasma Kit (Qiagen, Hilden, Germany) as per its manual instructions. TaqMan MicroRNA Reverse Transcription Kit (Thermo Fisher Scientific, Waltham, MA, USA) was used to prepare single-stranded cDNA in a reverse transcription reaction using 5 µg of RNA per the manufacturer’s protocol. The cycling condition for cDNA synthesis comprised incubating the reaction mixture at 25 °C for 10 min, 42 °C for 60 min, and 70 °C for 10 min. For each sample, analysis was performed using cDNA, MgCl_2_ (10 mM), Taq-polymerase (5 U/µl), PCR buffer, dNTP (10 mM), and a pair of specific primer (10 µM) in a 20-µl final reaction volume. The following were conditions of analysis: initial denaturation for 5 min at 95 °C followed by 40 cycles, annealing for 15 s at 95 °C, and extension for 20 s at 60 °C and final extension for 40 s at 72 °C. The following primers were used: miR-21, F: 5′-TAGCTTATCAGACTGATGTTGA-3′ and R: 5′-GAGGTATTCGCACTGGATACG-3′ and U6, F: 5′-CTCGCTTCGGCAGCACA-3′, and R: 5′-AACGCTTCACGAATTTGCGT-3′. The 2^−∆∆Ct^ comparative methodology was used to calculate the relative expression of the studied gene using U6 as the housekeeping gene (Livak and Schmittgen 2001).

#### Colorimetric assay

Determination of MDA and SOD content in sciatic nerve homogenates was performed using specific colorimetric kits obtained from Bio-diagnostic (Catalog No: MD2529 and SD2521, respectively, Cairo, Egypt) as per the kits’ instruction manual.

#### Enzyme-linked immunosorbent assay

Following manufacturers’ instructions, rat-specific enzyme-linked immunosorbent assay (ELISA) kits supplied by Mybiosource Inc. (Catalog No: MBS723034, MBS7230212, MBS2507393 and MBS825017, San Diego, CA, USA) were used to evaluate ATP, AMP, TNF-α, and IL-1β, respectively. At the same time, the rat ELISA kit obtained from Cusabio Technology LLC (Catalog No: CSB-EL002658RA, Wuhan, China) was used to measure beclin-1.

### Histopathologic examination

#### Light microscopy

The formalin-fixed sciatic nerve specimens were processed for paraffin embedding before being cut into 4-μm sections and then stained with hematoxylin–eosin (HE) staining and toluidine blue for light microscopic examination (Culling 2013). The extent of sciatic nerve fiber degeneration, Schwann cell loss, and inflammatory cells infiltration was used for grading the severity of the pathologic changes in the HE-stained sections. A 4-point scoring scale was used with 0, 1, 2, and 3 indicating no (0%), mild (1–25%), moderate (26–50%), and severe (> 50%) pathological changes, respectively (Ibrahim et al. 2020). Furthermore, the number of myelinated nerve fibers for each group was quantified by Leica QWin image analysis software (version 3; Leica Microsystems Ltd, Heerbrugg, Switzerland) using 5 random non-overlapping microscopic fields for each toluidine blue-stained section.

#### Immunohistochemistry

Paraffin-embedded sciatic nerves sections (4-μm thick) were used to evaluate MMP-2 and MMP-9 expression. Retrieved specimens were treated for 30 min at room temperature with 3% hydrogen peroxide/methanol, then washed with phosphate-buffered saline. Sections were treated with 10% goat blocking serum for 1 h at room temperature. Later, specimens were incubated with MMP-2 or MMP-9 rabbit monoclonal antibodies (1:100 dilution; Catalog No: MA5-13590 and MA5-14228, respectively, Thermo Fisher Scientific, Hanover, IL, USA) overnight at room temperature. After washing, sections were incubated with biotinylated secondary antibody (Dako, Glostrup, Denmark) and then with horseradish peroxidase-conjugated streptavidin for 60 min each at room temperature. Three additional washes were performed, then the reaction was visualized by 3,3′-diaminobenzidine tetrahydrochloride (DAB Substrate Kit, Vector Laboratories Inc., Burlingame, CA, USA). Slides were counterstained with hematoxylin, dehydrated, mounted, and examined by a light microscope. The area percentage of immunopositive cells to the total area of the microscopic field was analyzed by Leica QWin image analysis software (version 3; Leica Microsystems Ltd, Heerbrugg, Switzerland) at ×400 magnification. The analyses were conducted using 5 non-overlapping microscopic fields that have been randomly chosen from each section.

#### Electron microscopy

Briefly, sciatic nerves specimens were postfixed in osmium tetroxide (1%), dehydrated, embedded, cut into 1-μm sections. Following uranyl acetate and lead citrate staining, sciatic nerves’ ultrastructure was investigated and photographed using a transmission electron microscope (Hitachi H-300, Hitachi LTD., Tokyo, Japan). Then, they were analyzed by the Digimizer Image Analysis Software (version 5.4.4, © 2005–2020 MedCalc Software LTD) to measure the ratio of sciatic nerves’ axon to myelin sheath areas (Wang et al. 2018).

### Statistical analysis

GraphPad Prism software (version 8, San Diego, CA, USA) was used to analyze the results statistically. All datasets were subjected to the Shapiro test to check normality. The data was displayed as mean ± S.D and analyzed using one-way analysis of variance (ANOVA) and Tukey’s multiple comparison test. On the other hand, the pathological scoring was displayed as the median and range and analyzed by the nonparametric Mann–Whitney U test. The degree of significance for all statistical tests was set to p < 0.05.

## Results

### Effect of EMPA on body weight and blood glucose level in STZ-induced DPN in rats

**Fig. 2:**
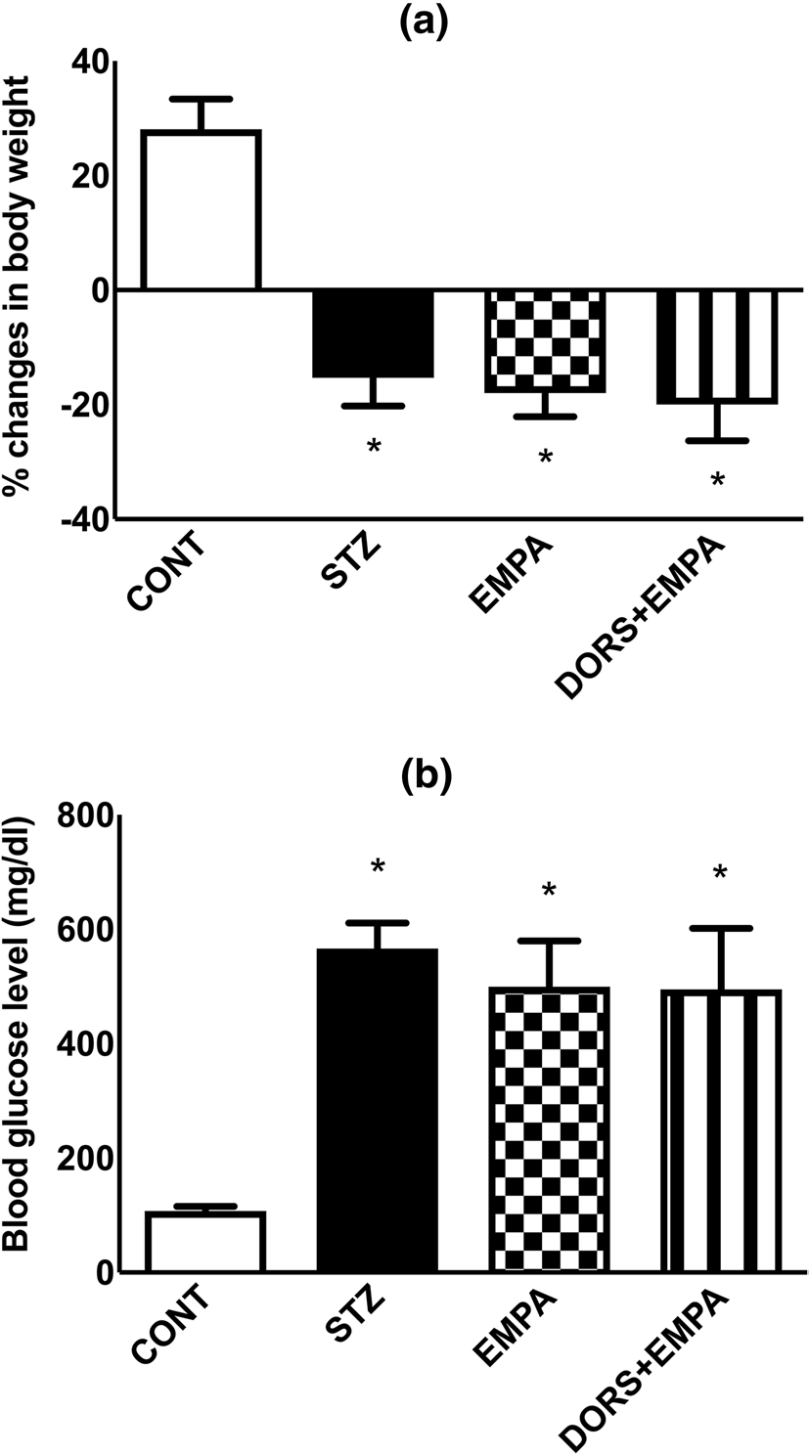
Effect of EMPA on body weight and blood glucose level in STZ-induced DPN in rats. Panels represent (**a**) % change in body weight and (**b**) fasting blood glucose level. Every bar with a vertical line displays the mean ± S.D (n = 10). (*) vs CONT, (@) vs STZ, (#) vs EMPA; P < 0.05. CONT: control; DPN: diabetic peripheral neuropathy; DORS: dorsomorphin; EMPA: empagliflozin; STZ: streptozotocin

Rats received STZ displayed a decline in body weight (Fig. 2a) along with an elevation in fasting blood glucose level (Fig. 2b) to 72% and 524%, respectively, comparable to the control group. Moreover, the administration of EMPA failed to improve the previous alterations. Similarly, DORS + EMPA-treated animals showed effects as observed in both STZ and EMPA groups.

### Effect of EMPA on motor and sensory performance in STZ-induced DPN in rats

**Fig. 3:**
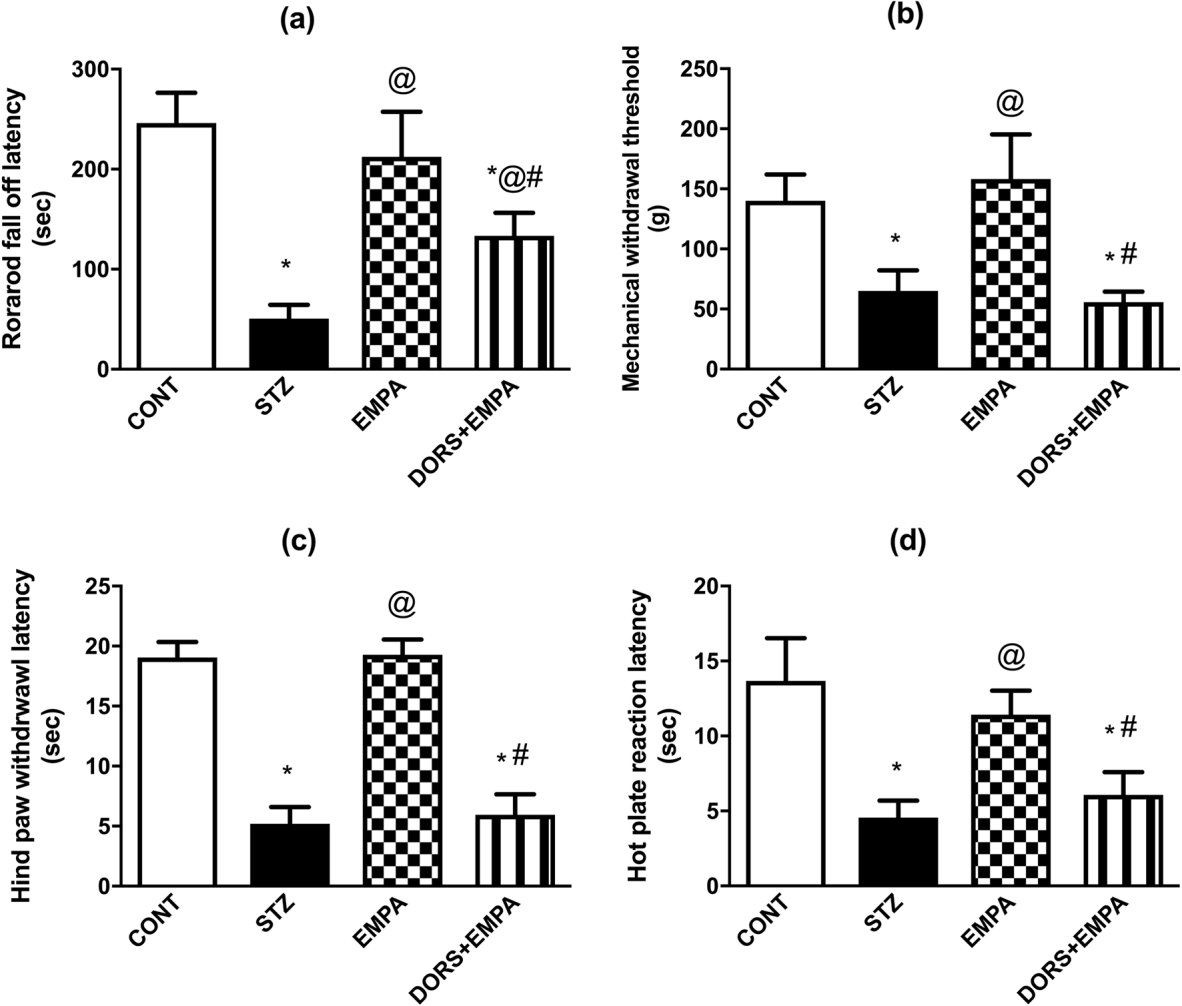
Effect of EMPA on motor and sensory performance in STZ-induced DPN in rats. Panels represent: (**a**) rotarod fall-off latency, (**b**) Randell-Selitto mechanical withdrawal threshold, (**c**) cold allodynia hind paw withdrawal latency, and (**d**) hot plate reaction latency. Every bar with a vertical line displays the mean ± SD (n = 8-10). (*) vs CONT, (@) vs STZ, (#) vs EMPA; P < 0.05. CONT: control; DPN: diabetic peripheral neuropathy; DORS: dorsomorphin; EMPA: empagliflozin; STZ: streptozotocin

The diabetic group displayed impairment in their motor and sensory performance during the behavioral assessments as manifested by decreased rotarod fall off latency (21%; Fig. 3a), Randell-Selitto mechanical withdrawal threshold (46%; Fig. 3b), cold allodynia hind paw withdrawal latency (27%; Fig. 3c) as well as hot plate reaction latency (33%; Fig. 3d), comparable to the control animals. The administration of EMPA almost normalized these changes, whereas DORS + EMPA-treated rats nearly abolished EMPA beneficial effects.

### Effect of EMPA on the histopathological alterations of the sciatic nerves in STZ-induced DPN in rats

**Fig. 4:**
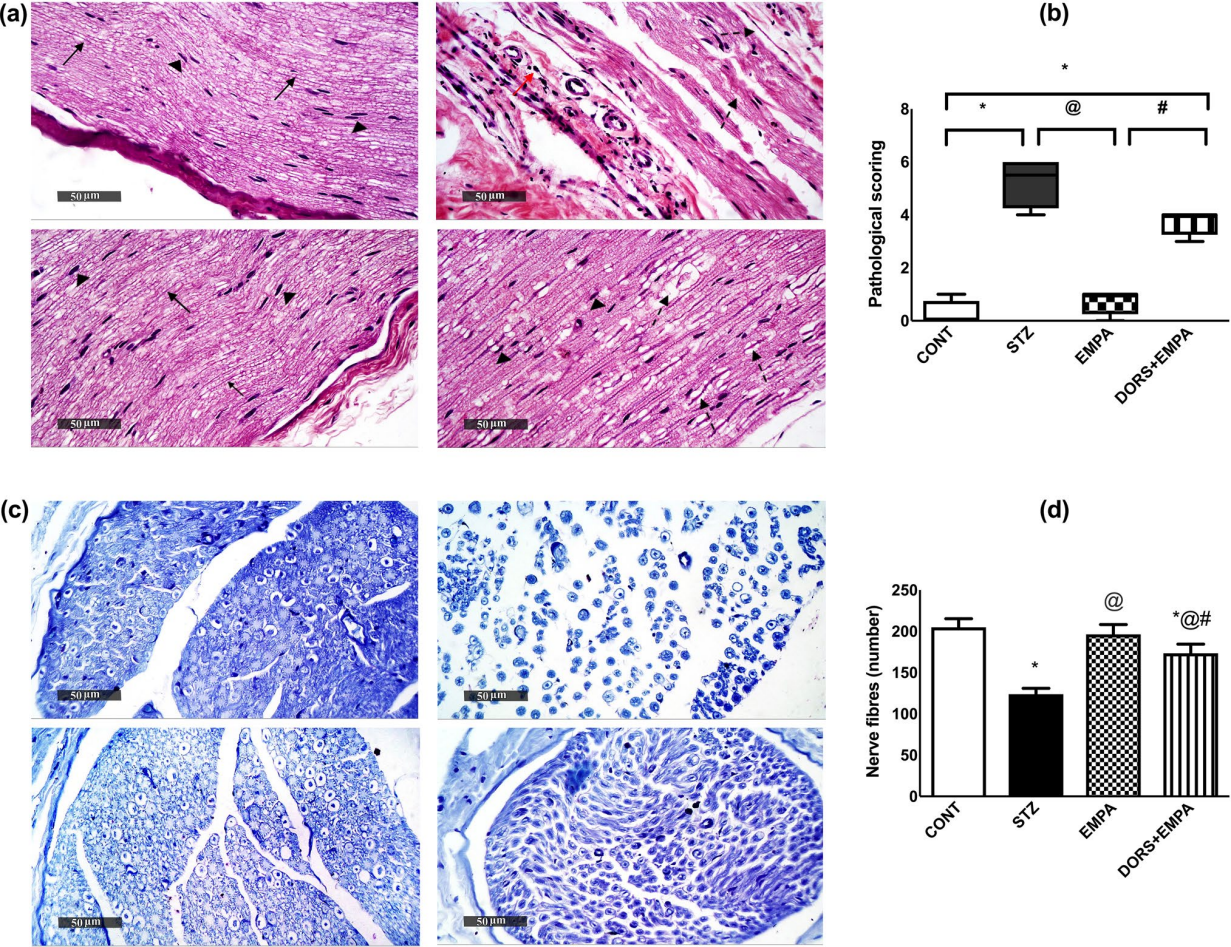
Effect of EMPA on the histopathological alterations of the sciatic nerves in STZ-induced DPN in rats. (**a**) Sections of sciatic nerves stained with hematoxylin and eosin. CONT section showing myelinated nerve fibers (arrow) and scattered Schwann cells (arrowhead). STZ section showing myelin sheath loss (dashed arrows), Schwann cells loss (arrowhead), and inflammatory cells (red arrow). EMPA section showing myelinated nerve fibers (arrow) and scattered Schwann cells (arrowhead). DORS+EMPA section showing edema (dashed arrows) and mild loss of Schwann cells (arrowhead) (Scale bar is 50 μm). (**b**) Pathological scoring. Every bar with a vertical line displays the median ± range (n= 4). (**c**) Sections of sciatic nerves stained with toluidine blue (Scale bar is 50 μm). (**d**) Nerve fibers' count. Every bar with a vertical line displays the mean ± SD (n=3). (*) vs CONT, (@) vs STZ, (#) vs EMPA; P < 0.05. CONT: control; DPN: diabetic peripheral neuropathy; DORS: dorsomorphin; EMPA: empagliflozin; STZ: streptozotocin

Microscopic examination of sciatic nerve sections (Fig. 4a) revealed that CONT animals showed apparently intact well-organized myelinated nerve fibers containing numerous Schwann cells and thin endoneurial connective tissue as well as perineural, epineural connective tissue sheath without abnormal cellular infiltrates. However, STZ rats showed multiple focal areas of axonopathies such as swelling of nerve fibers, endoneurial edema, occasional myelin sheath loss, reduced numbers of Schwann cells in some bundles, and severe epineural connective tissue mononuclear inflammatory cells infiltrates. Moreover, EMPA-treated samples showed almost amelioration of the previously mentioned changes. At the same time, DORS + EMPA sections showed similar but less severe damage than the STZ group. These effects were also represented by mitigation of the pathological score in EMPA-treated animals (Fig. 4b), compared to the STZ group. In addition, the STZ group exhibited a reduction in nerve fiber count (60.5%) compared to the CONT group (Fig. 4c, d). In comparison, the EMPA group showed an increased count by 58% compared to the STZ group. Moreover, the DORS + EMPA group partially improved the count of nerve fibers, comparable to the STZ group.

### Effect of EMPA on the micromorphological alterations of sciatic nerves in STZ-induced DPN in rats

**Fig. 5:**
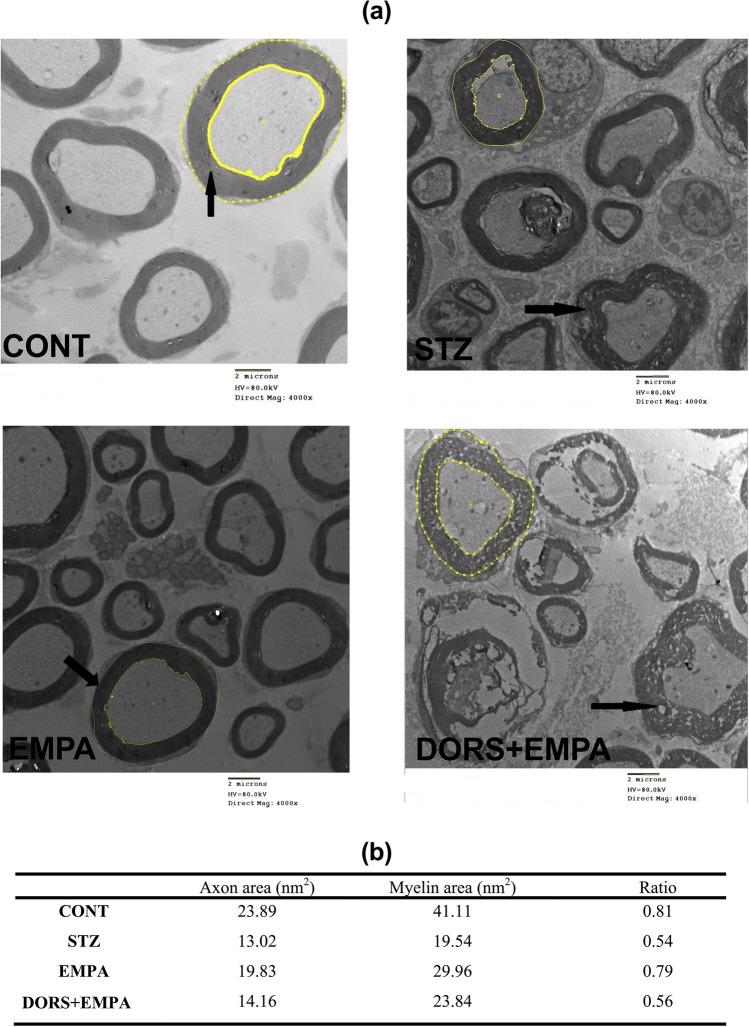
Effect of EMPA on the micromorphological alterations of the sciatic nerves in STZ-induced DPN in rats. (**a**) Sections of sciatic nerves stained with uranyl acetate and lead citrate. CONT section showing intact well-formed myelin sheath (arrow). STZ section showing deformed nerve fibers, axonal atrophy, and delamination of myelin lamellae (arrow). EMPA section showing almost well-formed axon with an intact myelin sheath. DORS+EMPA section showed axonal damage with areas of myelin loss (arrow) (Scale bar is 2 μm). (**b**) The ratio of the axon to myelin sheath areas. CONT: control; DORS: dorsomorphin; EMPA: empagliflozin; STZ: streptozotocin

Electron micrographs of rat sciatic nerves (Fig. 5a) displayed that myelinated nerve fibers of the CONT group exhibited complete and regular structures. STZ group revealed delamination of myelin lamellae, axonal atrophy, and deformed nerve fibers. However, the EMPA group displayed almost well-organized myelinated nerve fibers, while the DORS + EMPA group showed similarly deranged myelin sheaths and damaged nerve fibers as in the STZ group. Moreover, the axon to myelin sheath areas ratio in the sciatic nerve represents the extent of axonal atrophy and swelling of the myelin sheath (Fig. 5b). This ratio was improved in the EMPA-treated rats, comparable to either STZ or DORS + EMPA groups.

### Effect of EMPA on nerve conduction velocity in the sciatic nerves in STZ-induced DPN in rats

In Table 1, SNCV and MNCV were decreased by 18% and 11%, respectively, in STZ rats compared to normal rats. Though, both conduction velocities were normalized following treatment with EMPA. Nonetheless, administration of DORS + EMPA reduced SNCV insignificantly and MNCV significantly compared to the EMPA group.

**Table 1:** Effect of EMPA on sensory and motor nerve conduction velocities in the sciatic nerves in STZ-induced DPN in rats

	SNCV(m/s)	MNCV (m/s)
CONT	52.54 ± 2.10	48.34 ± 0.90
STZ	42.88 ± 3.58*	43.16 ± 1.03*
EMPA	49.71 ± 1.90^@^	50.49 ± 2.49^@^
DORS + EMPA	46.94 ± 3.63*	45.78 ± 2.72^#^

Results are displayed as mean ± SD (n = 6)

*CONT* control, *DPN* diabetic peripheral neuropathy, *DORS* dorsomorphin, *EMPA* empagliflozin, *MNCV* motor nerve conduction velocity, *SNCV* sensory nerve conduction velocity, *STZ* streptozotocin

*CONT, ^@^STZ, ^#^EMPA; *P* < 0.05

### Effect of EMPA on ATP/AMP ratio and AMPK expression in the sciatic nerves in STZ-induced DPN in rats

**Fig. 6:**
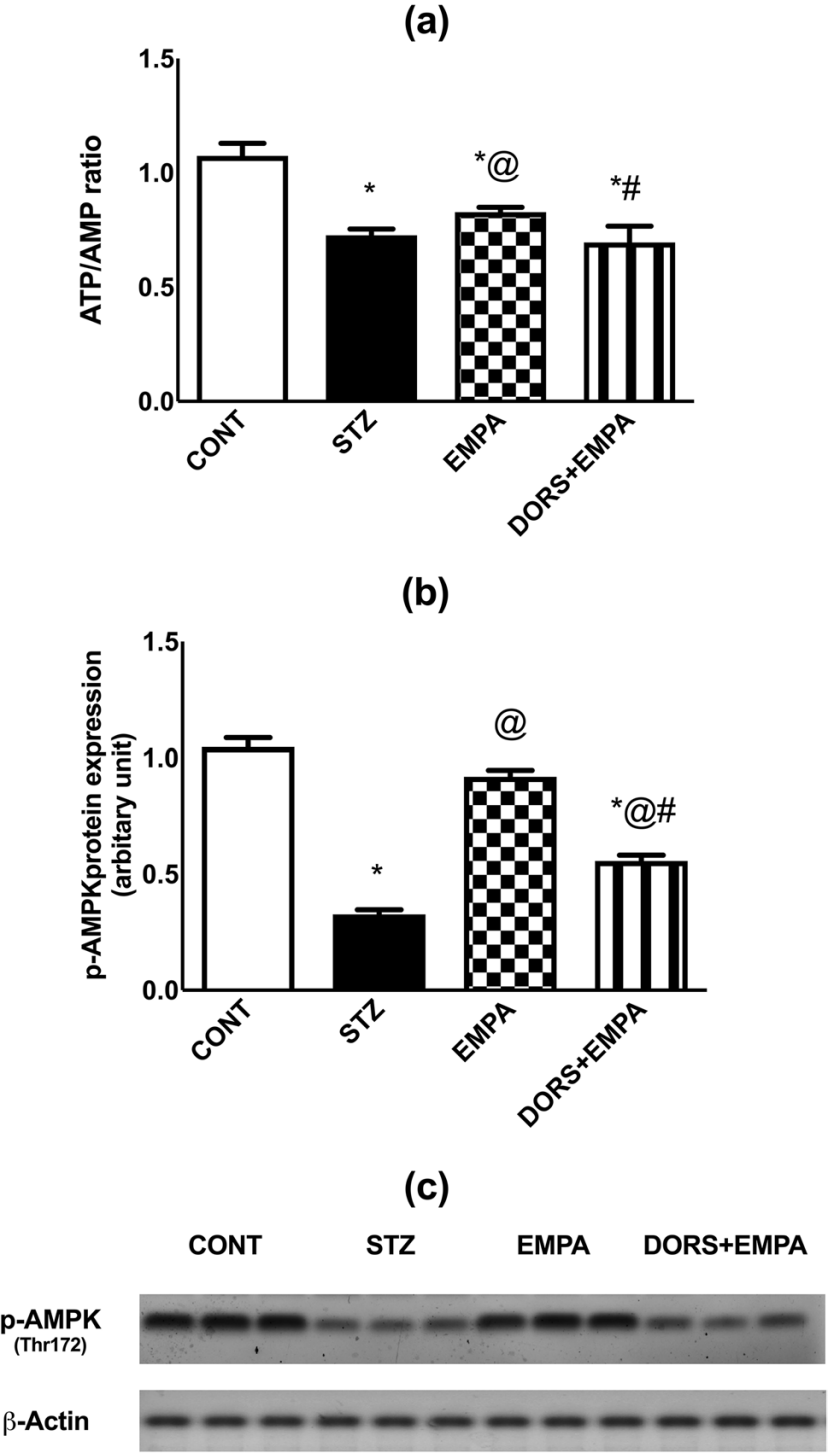
Effect of EMPA on ATP/AMP ratio and AMPK expression in the sciatic nerves in STZ-induced DPN in rats. Panels represent: (**a**) ATP/AMP ratio, (**b**) protein expression of p-AMPK (Thr172), and (**c**) corresponding p-AMPK western blotting bands. Results are displayed as mean ± SD (n= 3-6). (*) vs CONT, (@) vs STZ, (#) vs EMPA; P < 0.05. AMP: adenosine monophosphate; AMPK: adenosine monophosphate kinase; ATP: adenosine triphosphate; CONT: control; DPN: diabetic peripheral neuropathy; DORS: dorsomorphin; EMPA: empagliflozin; STZ: streptozotocin

The ATP/AMP ratio (Fig. 6a) was declined in the STZ group to 66%, related to the control animals, while EMPA-treated rats elevated this ratio by 14%, compared to the diabetic group. Contrariwise, DORS + EMPA-treated rats prevented EMPA-enhanced ATP/AMP ratio.

Additionally, EMPA stimulated the expression of p-AMPK (Fig. 6b, c) to reach 279%, compared to the STZ group that showed a decline in p-AMPK expression to 33%, compared to the normal animals. Moreover, DORS + EMPA administration partially reversed the EMPA action on p-AMPK expression.

### Effect of EMPA on NF-κB p65, p38 MAPK, ERK1/2, RECK, and miR-21 expressions in the sciatic nerves in STZ-induced DPN in rats

**Fig. 7:**
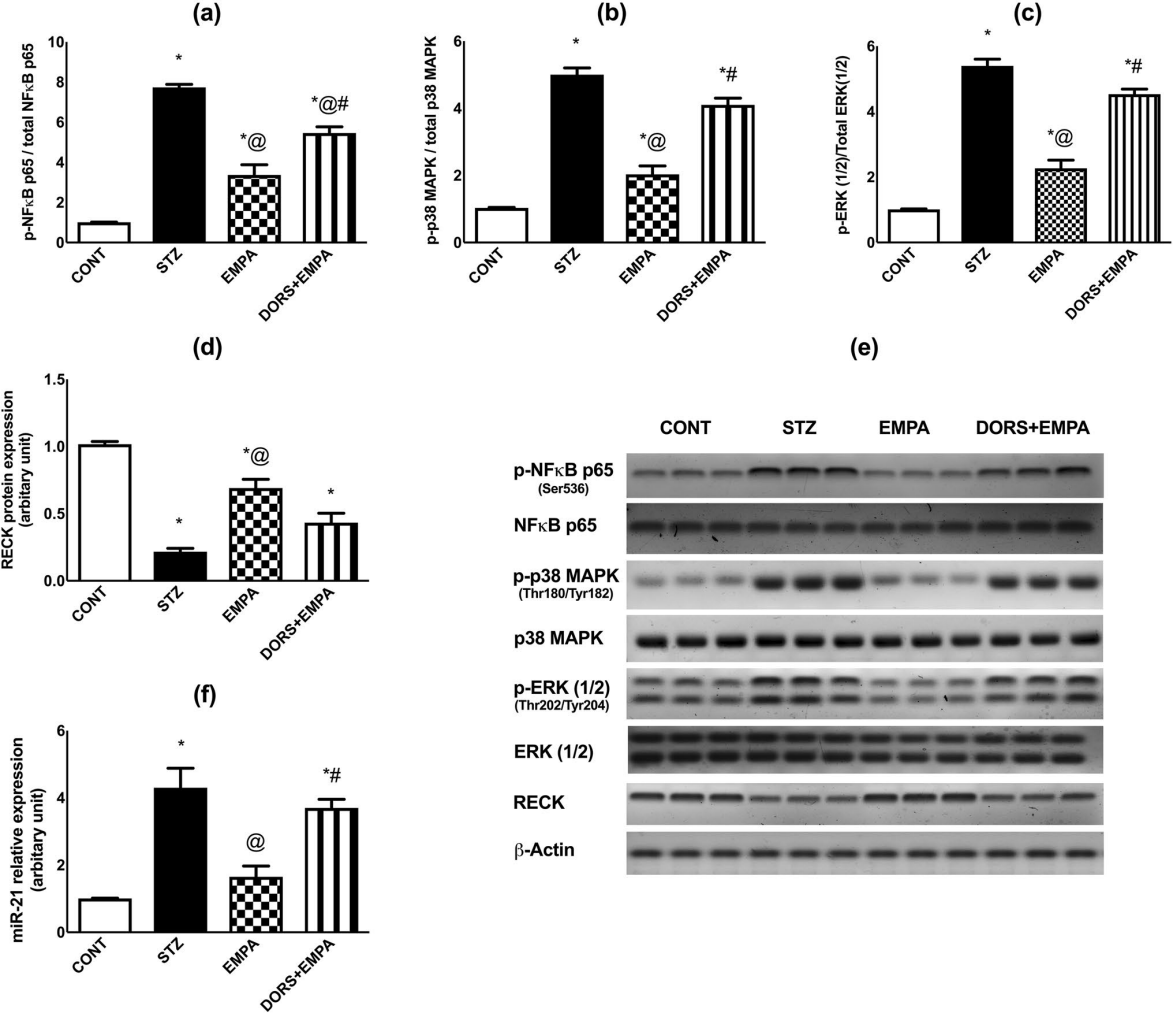
Effect of EMPA on NF-κB p65, p38 MAPK, ERK1/2, RECK, and miR-21 expressions in the sciatic nerves in STZ-induced DPN in rats. Panels represent protein expression of (**a**) p-NF-κB p65/total NF-κB p65, (**b**) p-p38 MAPK/total p38 MAPK, (**c**) p-ERK1/2/total ERK1/2, (**d**) RECK, and (**e**) corresponding western blotting bands along with relative expression of (f) miR-21. Results are displayed as mean ± SD (n = 3-4). (*) vs CONT, (@) vs STZ, (#) vs EMPA; P < 0.05. CONT: control; DPN: diabetic peripheral neuropathy; DORS: dorsomorphin; EMPA: empagliflozin; ERK: extracellular signal-regulated kinases; MAPK: mitogen-activated protein kinase; miR: micro-RNA; NF-κB: nuclear factor kappa-B; RECK: reversion-inducing cysteine-rich protein with Kazal motifs; STZ: streptozotocin

**Fig. 8:**
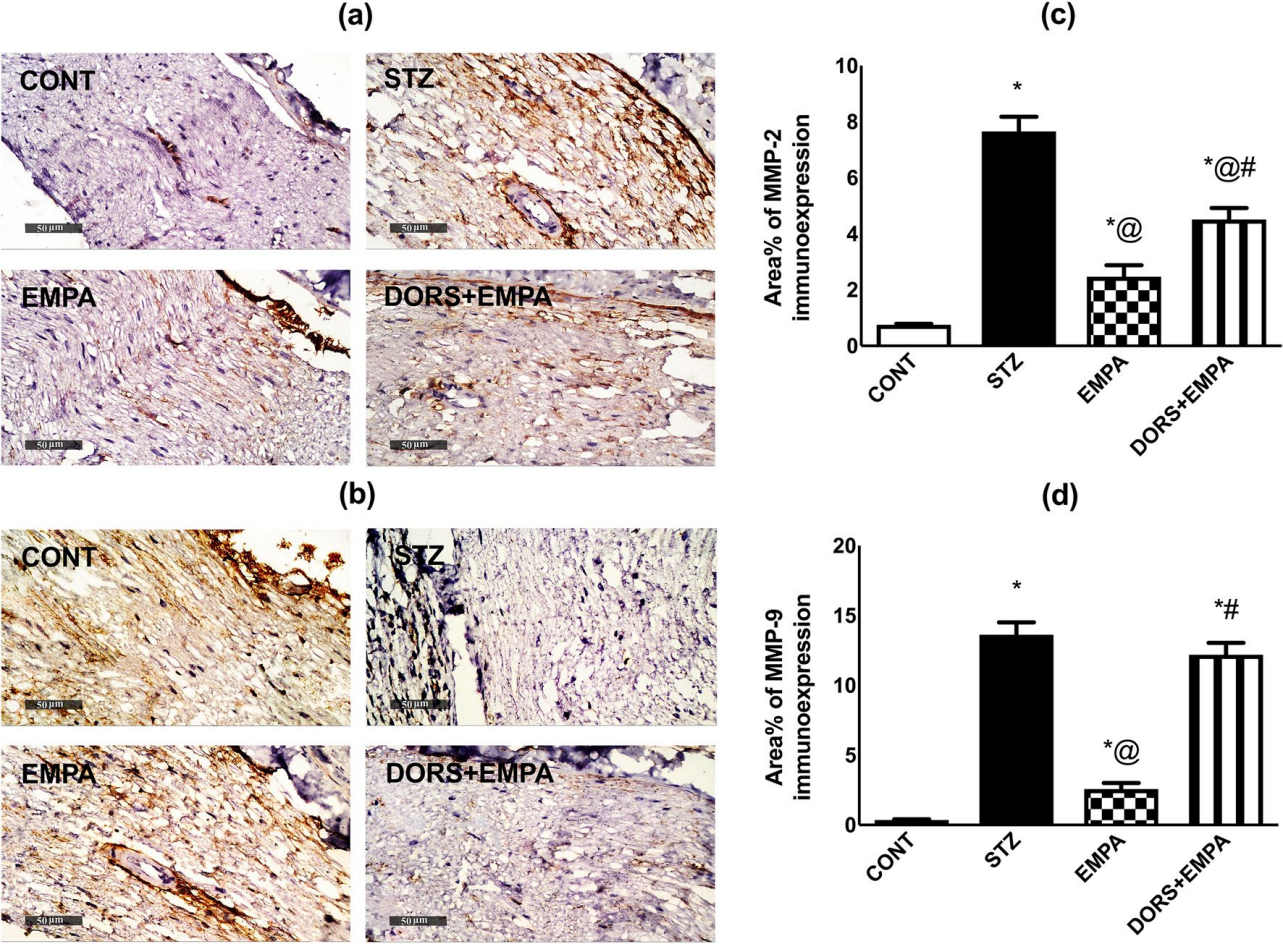
Effect of EMPA on MMP-2 and MMP-9 expression in the sciatic nerves in STZ-induced DPN in rats.Representative photomicrographs depicting (**a**) MMP-2 and (**b**) MMP-9 immunohistochemical staining in sciatic nerves (Scale bar is 50 μm). Panels represent the corresponding area % of (**c**) MMP-2 and (**d**) MMP-9 immunoexpression. Results are displayed as mean ± SD (n = 4). (*) vs CONT, (@) vs STZ, (#) vs EMPA; P < 0.05. CONT: control; DPN: diabetic peripheral neuropathy; DORS: dorsomorphin; EMPA: empagliflozin; MMP: metalloproteinase; STZ: streptozotocin

Administration of STZ elevated p-NF-κB p65/total NF-κB p65 (Fig. 7a, e), p-p38 MAPK/total p38 MAPK (Fig. 7b, e), p-ERK1/2/total ERK1/2 (Fig. 7c, e), and miR-21 expressions (Fig. 7f) to 8-, 5-, 5- and fourfold, respectively, related to the control animals. However, treatment with EMPA reduced the expression of the previous biomarkers by 56%, 60%, 57%, and 60%, respectively, compared to the diabetic rats. Coadministration of DORS and EMPA completely prevented the suppressive action of EMPA on p-p38 MAPK/total p38 MAPK and p-ERK1/2/total ERK1/2 as well as miR-21 expressions, and partially inhibited p-NF-κB p65 expression. Moreover, RECK expression (Fig. 7d, e) was downregulated in STZ-treated rats to 22%, comparable to the control group; however, administration of EMPA upregulated this expression by 213%, compared to the STZ group. Nevertheless, DORS + EMPA administration partially reduced RECK expression compared to EMPA-treated animals.

### Effect of EMPA on MMP-2 and MMP-9 expressions in the sciatic nerves in STZ-induced DPN in rats

The immunohistochemical expression of MMP-2 (Fig. 8a, c) and MMP-9 (Fig. 8b, d) were enhanced in the STZ-treated animals as indicated by high area % (10- and 39-fold, respectively) as well as the presence of brown staining, comparable to the normal rats. Furthermore, EMPA significantly downregulated the expression of these metalloproteinases compared to the diabetic animals. Nevertheless, the DORS + EMPA administration nearly blocked the effect of the EMPA group.

### Effect of EMPA on mTOR and ULK1 expressions and beclin-1 content in the sciatic nerves in STZ-induced DPN in rats

**Fig. 9:**
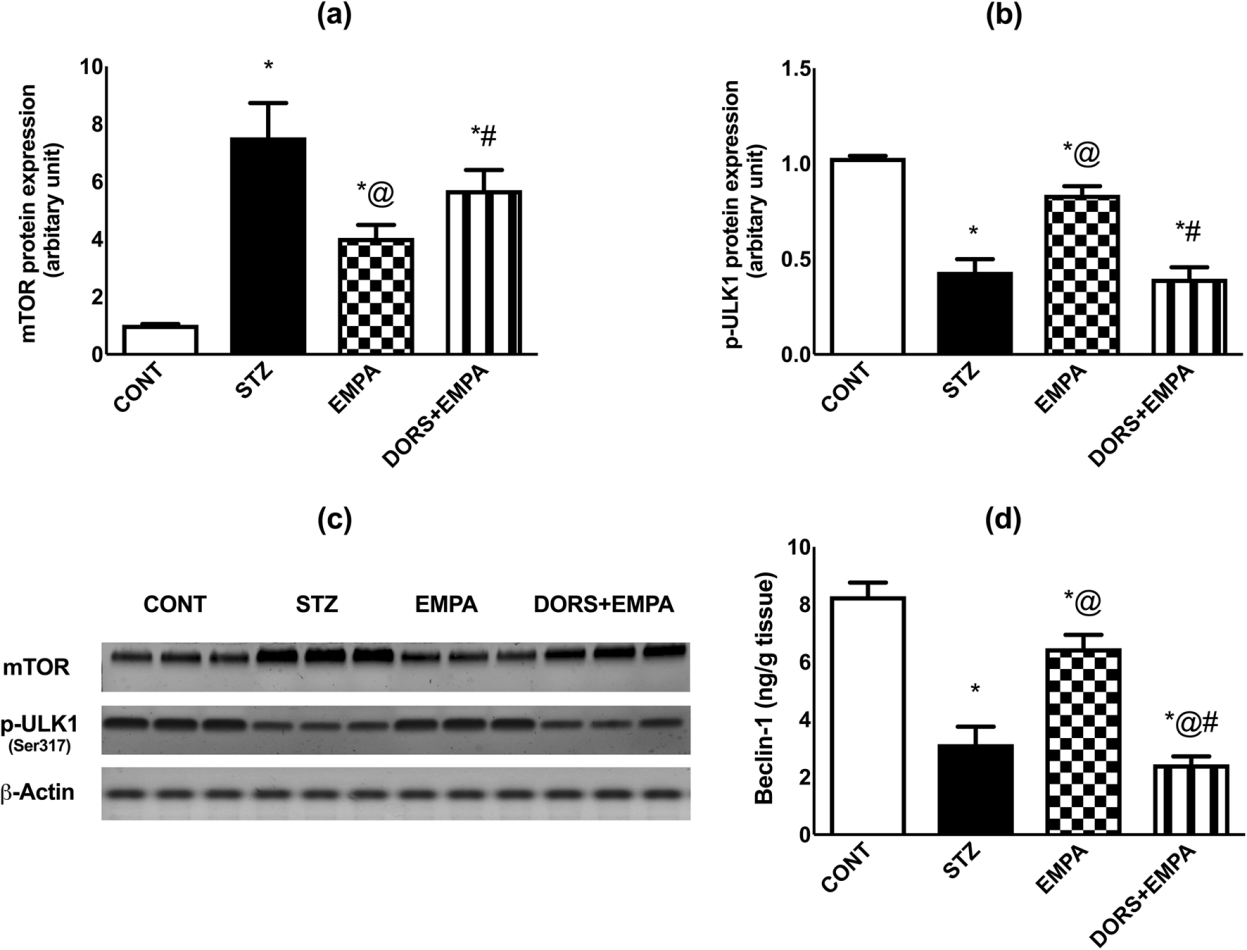
Effect of EMPA on mTOR and ULK1 expressions and beclin-1 content in the sciatic nerve in STZ-induced DPN in rats. Panels represent protein expression of (**a**) mTOR and (**b**) ULK1 (Ser317), (**c**) corresponding mTOR and ULK1 western blotting bands, and (**d**) beclin-1 content. Results are displayed as mean ± SD (n = 3-6). (*) vs CONT, (@) vs STZ, (#) vs EMPA; P < 0.05. Beclin-1: mammalian orthologue of yeast Atg6; CONT: control; DPN: diabetic peripheral neuropathy; DORS: dorsomorphin; EMPA: empagliflozin; mTOR: mammalian target of rapamycin; STZ: streptozotocin; ULK1: Unc-51 like autophagy activating kinase 1

STZ group showed enhanced expression of mTOR (Fig. 9a, c) to 7.5-fold, comparable to the control rats, while administration of EMPA reduced this expression by 47%, related to the STZ group. Instead, DORS + EMPA-treated rats showed insignificant changes from STZ rats. Furthermore, the relative expression of ULK1 (Fig. 9b, c) and the content of beclin-1 (Fig. 9d) were reduced in the STZ group to 43% and 37%, respectively, compared to the control animals, while administration of EMPA elevated ULK1 expression as well as beclin-1 content in the sciatic nerve by 95% and 110%, respectively when compared to the diabetic rats. Moreover, DORS + EMPA administration eradicated EMPA effects.

### Effect of EMPA on TNF-α, IL-1β and MDA contents and SOD activity in the sciatic nerves in STZ-induced DPN in rats

Levels of the cytokines, TNF-α and IL-1β, as well as the oxidative stress biomarker, MDA, have been elevated in the STZ group by 107%, 109%, and 170%, respectively, comparable to the control rats as shown in Table 2. At the same time, administration of EMPA ameliorated these effects by 7%, 5%, and 32%, comparable to the diabetic group. Conversely, DORS + EMPA-treated rats reversed EMPA effects to varying extents. On the other hand, administration of EMPA enhanced SOD activity by 50%, compared to the STZ group that showed depressed SOD activity to 52%, compared to the control animals (Table 2). In contrast, DORS and EMPA coadministration abolished the antioxidant potential of EMPA.

**Table 2:** Effect of EMPA on TNF-α, IL-1β and MDA contents and SOD activity in the sciatic nerves in STZ-induced DPN in rats

	TNF-α (pg/g tissue)	IL-1β (pg/g tissue)	MDA (μmol/g tissue)	SOD (U/g tissue)
CONT	405 ± 4.5	240 ± 3.5	5.6 ± 0.29	6.5 ± 0.92
STZ	435 ± 7.1*	261 ± 5.3*	9.5 ± 0.22*	3.4 ± 0.15*
EMPA	406 ± 4.3^@^	248 ± 3.1*^@^	6.5 ± 0.056*^@^	5.1 ± 0.16*^@^
DORS + EMPA	425 ± 4.5*^@#^	260 ± 6.2*^#^	7.1 ± 0.24*^@#^	3.4 ± 0.36*^#^

Results are displayed as mean ± SD (n = 6)

*CONT* control, *DPN* diabetic peripheral neuropathy, *DORS* dorsomorphin, *EMPA* empagliflozin, *IL-1β* interleukin-1β, *MDA* malondialdehyde, *STZ* streptozotocin, *SOD* superoxide dismutase, *TNF-α* tumor necrosis factor-α

*CONT, ^@^STZ, ^#^EMPA; *P* < 0.05

## Discussion

Diabetic peripheral neuropathy is a prevalent diabetic complication manifested by spontaneous allodynia and hyperalgesia in about half of the patients (Jensen and Finnerup 2014). In the current work, apart from its anti-hyperglycemic effect, EMPA alleviated STZ-induced DPN in rats via amelioration of nociceptive threshold as indicated by an improved response to thermal stimuli in accordance with the work of Lee et al. (2018). The hot plate test showed hyper-responsiveness in diabetic animals, as displayed in earlier investigations (Mabley et al. 2003; Zan et al. 2017). Moreover, EMPA administration modulated hyperalgesia and cold allodynia, implicated with neuropathic pain elicited by peripheral nerves injury (Allchorne et al. 2005). Likewise, Lee et al. (2018) displayed that EMPA averted the hypersensitivity of diabetic rats in the analgesimeter test. Additionally, EMPA amended behavioral tests related to nociceptive and pain threshold and enhanced the motor coordination performance on the rotarod test, which has been impaired in STZ-induced DPN in experimental animals (Sharma et al. 2012; Abdelkader et al. 2022). EMPA-ameliorated DPN was confirmed by improved sciatic nerve histopathological structure, according to Lee et al. (2018). In addition to myelin preservation detected by the electron microscope, such alterations were observed in neuropathy caused by STZ in rats (Zangiabadi et al. 2011, 2014; Lee et al. 2018; Abdelkader et al. 2022).

In the current work, although EMPA neither maintained blood glucose level nor body weight, it halted the oxidative stress through stimulating SOD activity and reducing lipid peroxidation. Likewise, EMPA increased p-AMPK, improved ATP/AMP ratio, and inhibited p-p38 MAPK/p-ERK1/2/p-NF-κB p65/IL-1β and TNF-α signaling. Noteworthy, EMPA hindered miR-21 and enhanced RECK expression, reducing matrix metalloproteinase expression.

In the present work, EMPA administration at a dose of 3 mg/kg/day failed to achieve either glycemic control or maintained body weight. This effect agreed with the work of Lee et al. (2018), which displayed that the EMPA-mediated anti-hyperglycemic effect was dose-dependent. Meanwhile, the continuous loss of body weight seen herein might be attributed to the mild osmotic diuresis and calories loss in the urine induced by EMPA (Kovacs et al. 2014). Therefore, the potential effect of EMPA to combat DPN seen herein may be attributed to glucose-independent mechanisms, which was in accordance with a preliminary study that documented the neuro- and nephroprotective effects of EMPA in diabetic animals of type 1 (Lee et al. 2018).

This work elaborates the possible molecular mechanisms underlying the protective effect of EMPA against DPN. Persistent hyperglycemia can lead to neuronal apoptosis via deteriorated electron transport chain leading to disrupted ATP production (Vincent et al. 2002), as shown in experimental animals with STZ-induced neuropathy (Najafi et al. 2017). This event is accompanied by reduced AMPK phosphorylation, in accordance with earlier in vitro and in vivo experiments using human renal proximal tubular cells (hRPTCs) incubated in the hyperglycemic environment and STZ-induced diabetic mice, respectively (Lee et al. 2019). Consequentially, impaired bioenergetics occurred in neurons, reducing axonal health via interfering with axonal plasticity (Bernstein and Bamburg 2003). Herein, EMPA counteracted energy deprivation and upregulated AMPK phosphorylation. In accordance, it was reported that EMPA increased ATP level both in vivo in the hearts of diabetic mice (Verma et al. 2018) and lipopolysaccharide-treated mice as well as in vitro in lipopolysaccharide-treated macrophages and cardiomyocytes (Koyani et al. 2020). Moreover, Koyani et al. (2020) revealed that energy level was restored after EMPA-mediated AMPK activation. EMPA mostly increases ATP production via acetyl coenzyme A carboxylase (ACC) phosphorylation, a downstream target molecule of AMPK. AMPK/ACC pathway is responsible for improving energy metabolism via increasing fatty acid oxidation and ATP generation (Lu et al. 2020). AMPK phosphorylation is implicated in the amelioration of DPN by targeting various signaling molecules. This effect is in accordance with previous reports that documented the ability of EMPA to activate this axis in kidneys of diabetic mice (Lee et al. 2019), hearts of lipopolysaccharide-treated mice (Koyani et al. 2020), and a cardiac ischemia model in mice (Lu et al. 2020) as well as in healthy conditions in vivo and in vitro (Koyani et al. 2020). Noteworthy, AMPK activation by anti-hyperglycemic drugs has been implicated to a certain extent in their protective effects, as seen in metformin-induced alleviation of cardiomyopathy in OVE26 diabetic mice (Xie et al. 2011). This effect might be explained by the inhibitory action of metformin on the respiratory chain complex I, causing a decline in intracellular ATP level, thus elevating AMP/ATP ratio and activating AMPK (Owen et al. 2000; Zhou et al. 2001). However, other studies reported the potential of metformin to activate AMPK apart from disrupting the AMP/ATP ratio (Hawley et al. 2002; Bergheim et al. 2006). Similarly, the SGLT-2 inhibitor canagliflozin inhibited complex I in human embryonic kidney and liver cells to activate AMPK, thus reducing inflammation (Hawley et al. 2016). Regarding EMPA, it restored AMP/ATP ratio, which activated AMPK, thus preserving cardiac and mitochondrial function in mice cardiomyocytes. Furthermore, EMPA can either phosphorylate or prolong AMPK activation (Zhou et al. 2018). Also, Lu et al. (2020) showed that EMPA could activate AMPK by stimulating its upstream activator, liver kinase B1(LKB1). In addition, Ibrahim et al. (2022) reported that the SGLT-2 inhibitor dapagliflozin directly phosphorylated LKB1, leading to increased hippocampal expression of p-AMPK in the ovariectomized/D-galactose rat model of Alzheimer’s disease.

In the current work, EMPA mediated-sciatic AMPK-activation suppressed MAPK signaling that played a crucial part in neuropathic pain via peripheral nociceptors sensitization and reduced plasticity-related proteins (Obata and Noguchi 2004; Anand et al. 2011). As observed herein, previous studies reported that p38 MAPK phosphorylation at Tyr182 and Thr180 as well as phosphorylation of ERK at Thr202 and Tyr204 were predominant in experimental models of neuropathy (Jin et al. 2003; Schäfers et al. 2003; Tsuda et al. 2004; Zhuang et al. 2005). In addition, they reported that using p38 MAPK or ERK inhibitors ameliorated such neuropathic insult. Of note, AMPK activation negatively regulates p38 MAPK and ERK1/2 via phosphorylating the adaptor proteins that regulate receptor tyrosine kinases as well as inhibiting small GTPases, which are upstream activators for the MAPK signaling (Asiedu et al. 2016). In context, EMPA-induced AMPK activation inhibited p38 MAPK and ERK1/2 activation in hepatocytes of mice intoxicated with carbon tetrachloride.

In the current study, reduced MNCV and SNCV observed in STZ-injected rats could be mediated through polyol pathway-stimulated p38 MAPK signaling, resulting in NCV deficits (Agthong and Tomlinson 2002). EMPA administration improved both MNCV and SNCV in harmony with Eid et al. (2020) results, indicating that EMPA partially increased MNCV and SNCV in the DM type 1 mouse model. Noteworthy, EMPA may modulate NCV via downregulation of sciatic p38 MAPK, and this was in alignment with an earlier work displaying the in vitro repressing effect of EMPA on p38 MAPK (Das et al. 2020)*.* Furthermore, EMPA inhibitory effect on p38 MAPK may be through oxidative stress suppression seen in the current study and represented by reduced lipid peroxidation and enhanced SOD activity; such event is in line with Das et al. (2020) and Eid et al. (2020), who documented antioxidant capacity of EMPA in different models. Indeed, increased ROS generation due to hyperglycemia resulted in decreased mitochondrial membrane potential with subsequent ATP depletion, ultimately causing attenuation of nerve conduction ability (Visnagri et al. 2012).

Likewise, after nerve injury, p38 MAPK/ERK1/2 upregulated the transcription factor NF-κB p65 as shown herein (Ji and Suter 2007; Milligan and Watkins 2009) as well as enhanced related downstream mediators viz*.* IL-1β and TNF-α (Krakauer 2004). These inflammatory mediators stimulate the nociceptive neurons developing pain hypersensitivity (Ji and Suter 2007). Herein, EMPA ameliorated the deleterious effect of such inflammatory mediators through suppressing p-p38 MAPK/p-ERK1/2/p-NF-κB p65 expression, and this coincides with earlier studies displaying the potential impact of EMPA to treat inflammatory kidney diseases as well as hepatic inflammation and fibrosis via inhibition of this axis (Das et al. 2020; Abdelhamid et al. 2021).

Noteworthy, EMPA’s inhibitory effect on NF-κB p65/TNF-α and IL-1β might be linked to the activation of AMPK reported in the current work. In parallel, a previous study displayed the connection between this signaling pathway where AMPK activation downregulated NF-κB in the complete Freund’s adjuvant-induced inflamed skin tissues, thus decreasing the inflammatory mediators resulting in reduced pain sensation (Xiang et al. 2019).

In the current work, mTOR contributes to DPN development in STZ receiving animals due to hyperglycemia-reduced AMPK activation or -enhanced TNF-α, which were formerly documented (He et al. 2019). Also, mTOR-related DPN could be explained by reducing the adapter protein APPL1, a crucial protein in synaptic plasticity, or triggering mTOR activation. Thereby, inducing mechanical and thermal hyperalgesia could be stimulated by synapsin II-mediated neurite outgrowth, participating in hyperalgesia (He et al. 2019). On the contrary, this hyperalgesia was ameliorated through EMPA administration via the modulatory effect on AMPK and/or EMPA mediated anti-inflammatory properties, as seen herein. Recently, Sun et al. (2020) has reported the impact of EMPA in alleviating AMPK/mTOR cue in obesity-related cardiac dysfunction in mice. Noteworthy, by an alternative mechanism, mTOR inhibition could ameliorate DPN through induction of autophagy, removing any damaged cellular components and permitting cells to correct their metabolic demands, hence increasing myelin sheath thickness and the myelinated axons (Liu et al. 2018). Downregulation of mTOR stimulated the phosphorylation of ULK1, consequentially activation of beclin-1 to alleviate pain via enhancing autophagy (Russell et al. 2013). This effect was parallel with a study reporting that EMPA ameliorated diabetic tubulopathy by controlling autophagy (Lee et al. 2019).

Of note, in this work, MMP-2 and MMP-9 expression in the sciatic nerves was upregulated in the diabetic group, which was in accordance with Moustafa et al. (2018b), who documented alleviation of DPN via controlling extracellular matrix (ECM) remodeling. These metalloproteinases were involved in DPN through degrading ECM components implicated in arteries abnormalities leading to ischemia and neural death (Singh et al. 2011). This elevation may be related to enhanced oxidative stress/p38 MAPK/NF-κB p65 that affects transcriptional regulation of MMPs as reported in photoaging and photocarcinogenesis (Pittayapruek et al. 2016). Additionally, the observed modulatory effect of EMPA on MMPs expression could be attributed to enhancing RECK expression, an endogenous MMP inhibitor, via inhibiting oxidative stress/p-NF-κB p65 and p-p38 MAPK/miR-21 shown herein and goes align with Das et al. (2020), who suggested this renoprotective pathway of EMPA. Moreover, miR-21 stimulation was associated with reduced mechanical thresholds and heat withdrawal latencies as documented in spared nerve injury to contribute to neuropathic pain (Karl et al. 2017). Such effect was reversed with EMPA treatment.

Collectively, all beneficial effects produced by EMPA were almost abolished by using DORSO, an AMPK antagonist, which emphasizes the significance of AMPK involvement in DPN management as a promising contender.

In conclusion, the SGLT-2 inhibitor, EMPA, may be a promising candidate not only for DPN but also in neuropathic pain generally. It improved DPN and its associated symptoms apart from EMPA anti-diabetic effect. EMPA neuroprotective effects could be mediated via modulation of the signaling pathways: AMPK/p38 MAPK/ERK1/2/NF-κB p65/inflammatory mediators, AMPK/p38 MAPK/miR-21/RECK/metalloproteinases, or AMPK/mTOR to give credit to AMPK activation in combating DPN. This study has some limitations that should be considered. The current study explored the neuroprotective impact of EMPA against peripheral neuropathy in the STZ-induced DM type 2 model in rats, and more experiments in different pain models are needed to verify its efficacy against other types of neuropathic pain. In addition, this study examined EMPA’s potential mechanistic pathway in curbing DPN through targeting AMPK. Thus, additional in vitro and/or in vivo experiments are necessary to thoroughly investigate the exact underlying mechanisms using different blockers for the possible downstream pathways. On the other hand, the current study is the first to report that EMPA is effective against DPN in the rat model of DM type 2, along with exploring some possible underlying mediators and signaling pathways. A few recent studies revealed that EMPA ameliorated peripheral neuropathy in DM type 1 experimental models without mechanistic insights, though SGLT-2 inhibitors have not yet been approved for type 1 DM (Lee et al. 2018; Eid et al. 2020).

## Supplementary Information

Below is the link to the electronic supplementary material.

Supplementary file1 (DOCX 47 kb)

Supplementary file2 (PDF 8055 kb)
