# Supplementary material for: Empagliflozin mitigates type 2 diabetes-associated peripheral neuropathy: a glucose-independent effect through AMPK signaling
Source: Arch Pharm Res. 2022 Jun 29;45(7):475–93. doi: 10.1007/s12272-022-01391-5 (PMC9325846; doi:10.1007/s12272-022-01391-5)
Supplement: Supplementary file 1 — Supplementary file1 (DOCX 47 kb) [file 12272_2022_1391_MOESM1_ESM.docx]

**Supplementary experiment:**

**Animals**

Fourteen adult male Wistar rats (170-220 g) were purchased from the National Research Center (Giza, Egypt), then animals were allowed to accommodate in the animal facility of the Faculty of Pharmacy, Cairo University (Cairo, Egypt) for a week. They were kept under temperature of 22 ± 2 °C, relative humidity of 60 ± 10%, and a 12 h light/dark cycle. All rats had free access to tap water and standard laboratory chow.

**Experimental Design**

Rats were allocated in two groups as the following:

**Group I** (n=6) that served as the control group

**Group II** (STZ group; n=8) that represented the diabetic model group

Animals were exposed to overnight fasting, then to induce T2DM, the STZ group received nicotinamide (50 mg/kg; i.p) then after 15 min, animals received STZ (52.5 mg/kg; i.p). Moreover, following STZ injection, tap wate was replaced by 5% glucose solution for 24 h to reduce hypoglycemic shock-induced mortality. Two days post STZ challenge, blood samples were withdrawn from the tail vein of each rat under local anesthesia to evaluate blood glucose level using glucometer (ACCU-Check; Roche Molecular Biochemicals, Indianapolis, IN, USA). The hyperglycemia criteria included rats with BGL equal or more than 200 mg/dL. Thereafter, animals were anesthetized, and blood samples were collected via the retro-orbital plexus followed by serum separation by centrifugion at 3000 g for 10 min for determination of insulin level.

**Determination of insulin level**

Serum insulin level was determined using rat-specific ELISA kit (RayBio, Norcross, GA, USA, Catalog #: ELR-Insulin) according to the manufacturer instructions.

**Results**

*

**Supplementary Fig. 1 Effect of STZ on serum insulin level in STZ-induced DPN in rats. Each** bar with a vertical line represents the mean ± S.D (n = 6). (*) *vs* Control; *P*< 0.05. CONT: control; DPN: diabetic peripheral neuropathy; STZ: streptozotocin

The significance of differences was assessed by the Unpaired Student’s t test using GraphPad Prism software (version 8, San Diego, CA, USA).

**Conclusion**

As insulin concentration was detected in STZ group but at lower level compared to the control one, this indicates that there is a defect (not complete) in pancreatic insulin secretion, which represents type II DM.
