# Supplementary figures and images for: Empagliflozin mitigates type 2 diabetes-associated peripheral neuropathy: a glucose-independent effect through AMPK signaling
Source: Arch Pharm Res. 2022 Jun 29;45(7):475–93. doi: 10.1007/s12272-022-01391-5 (PMC9325846; doi:10.1007/s12272-022-01391-5)

pAMPK

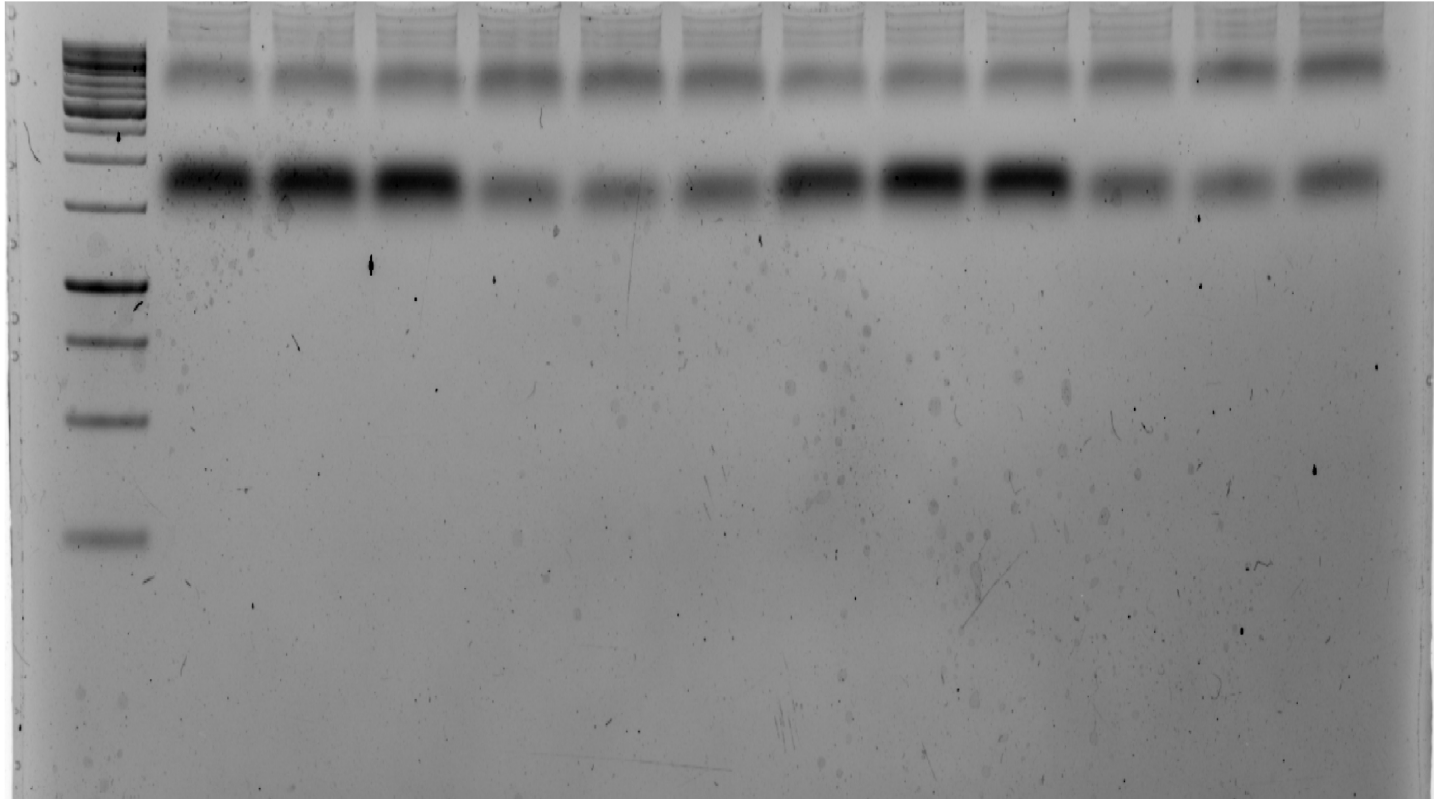

P-ULK1

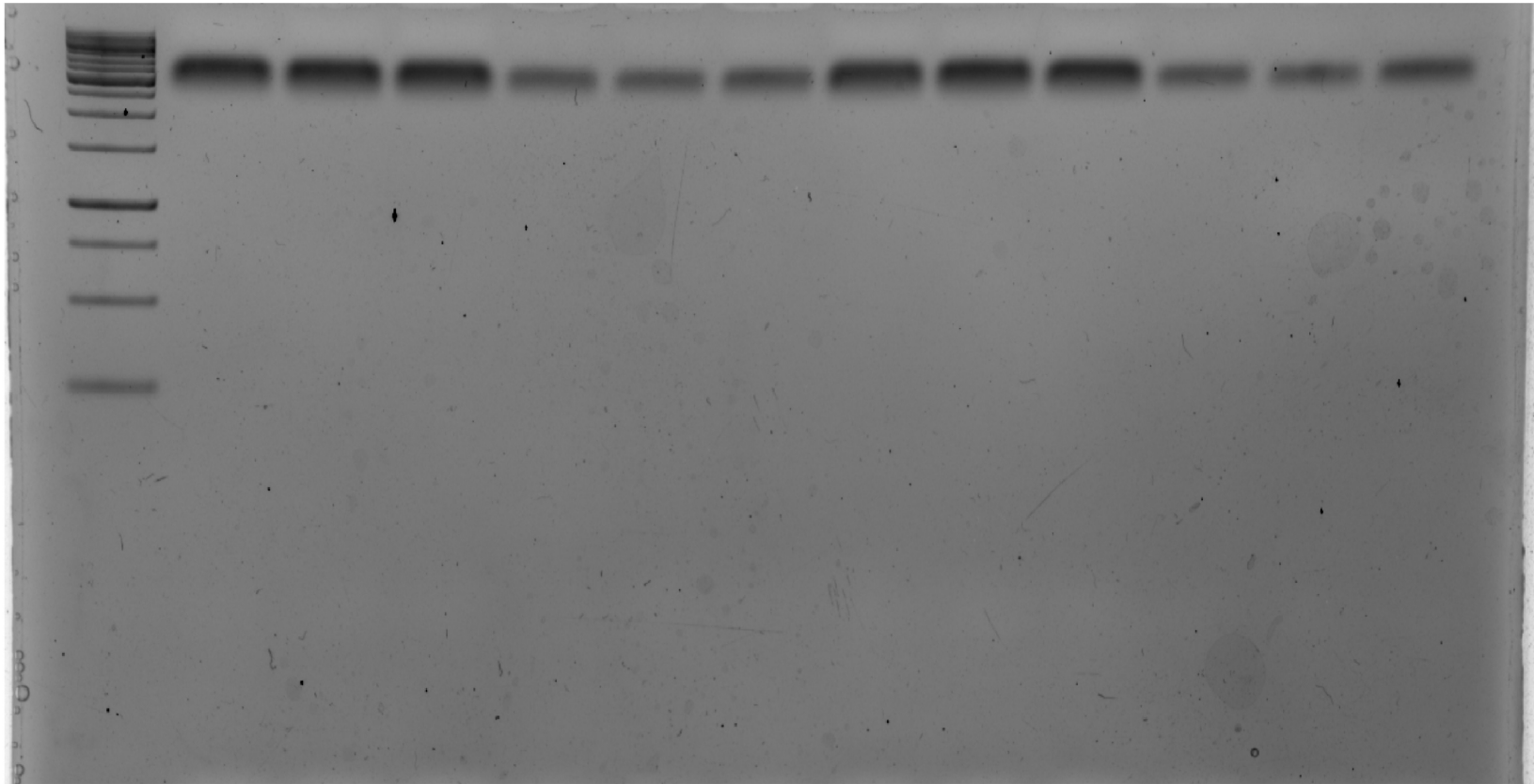

RECK

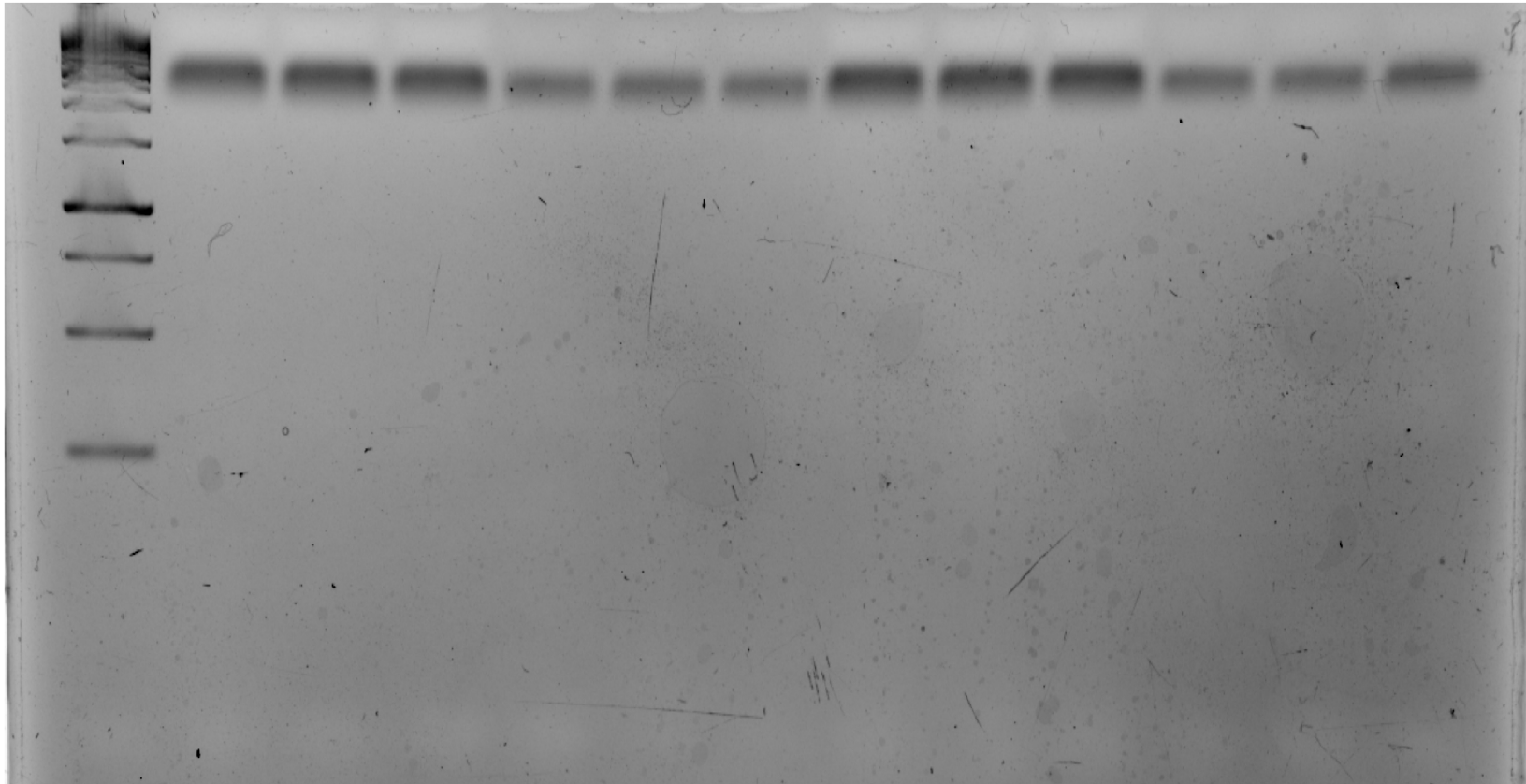

NFkB p65

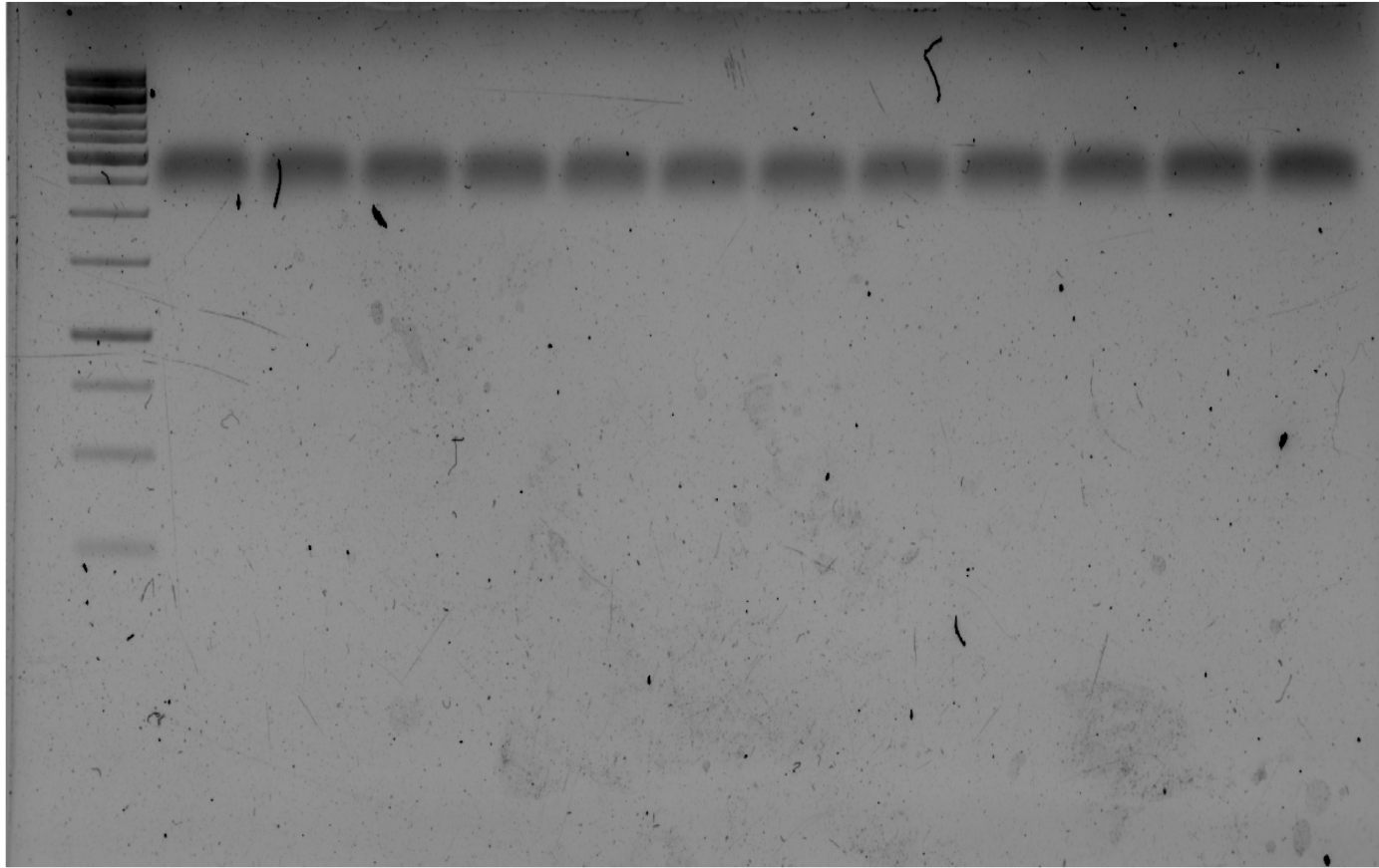

P-NFkB p65

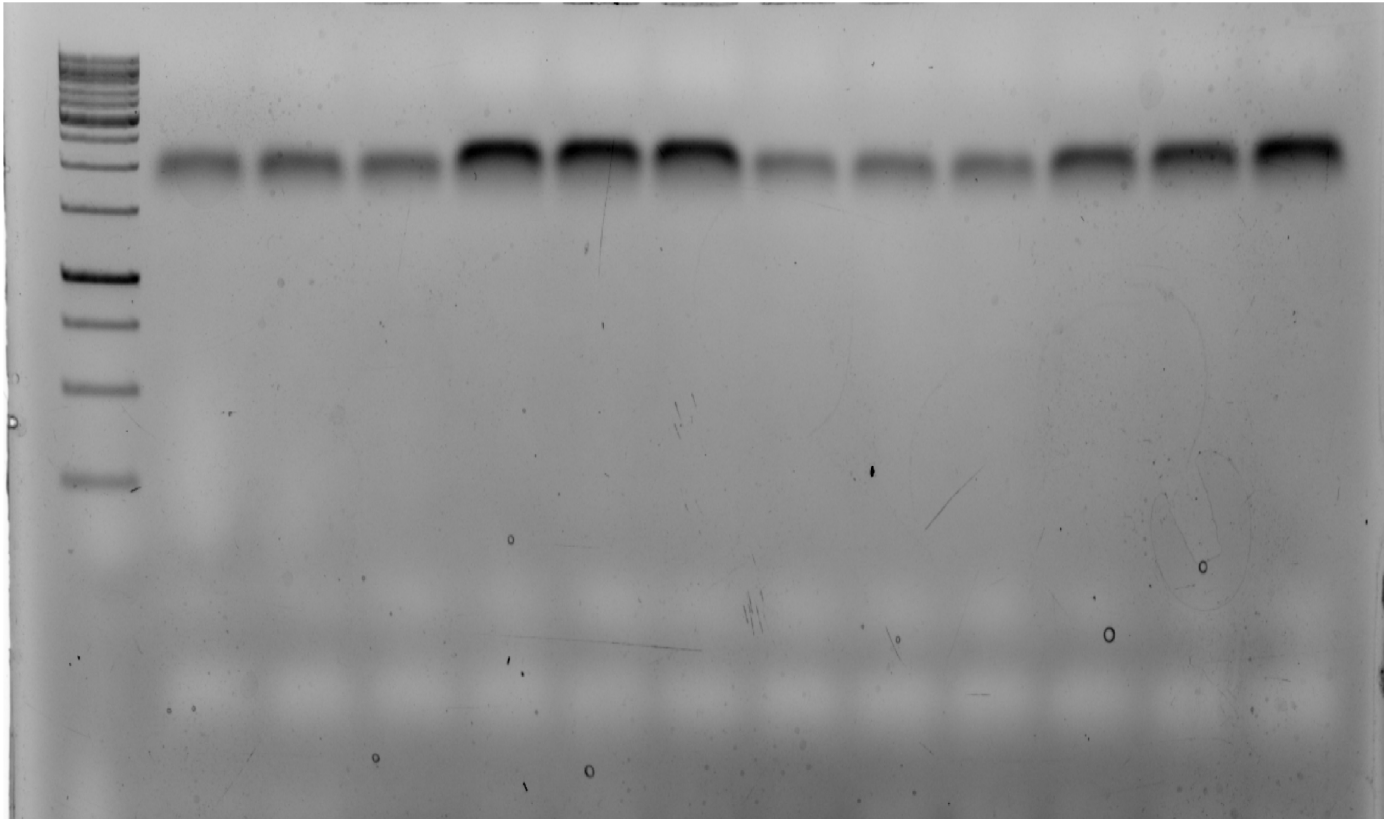

P-mTOR

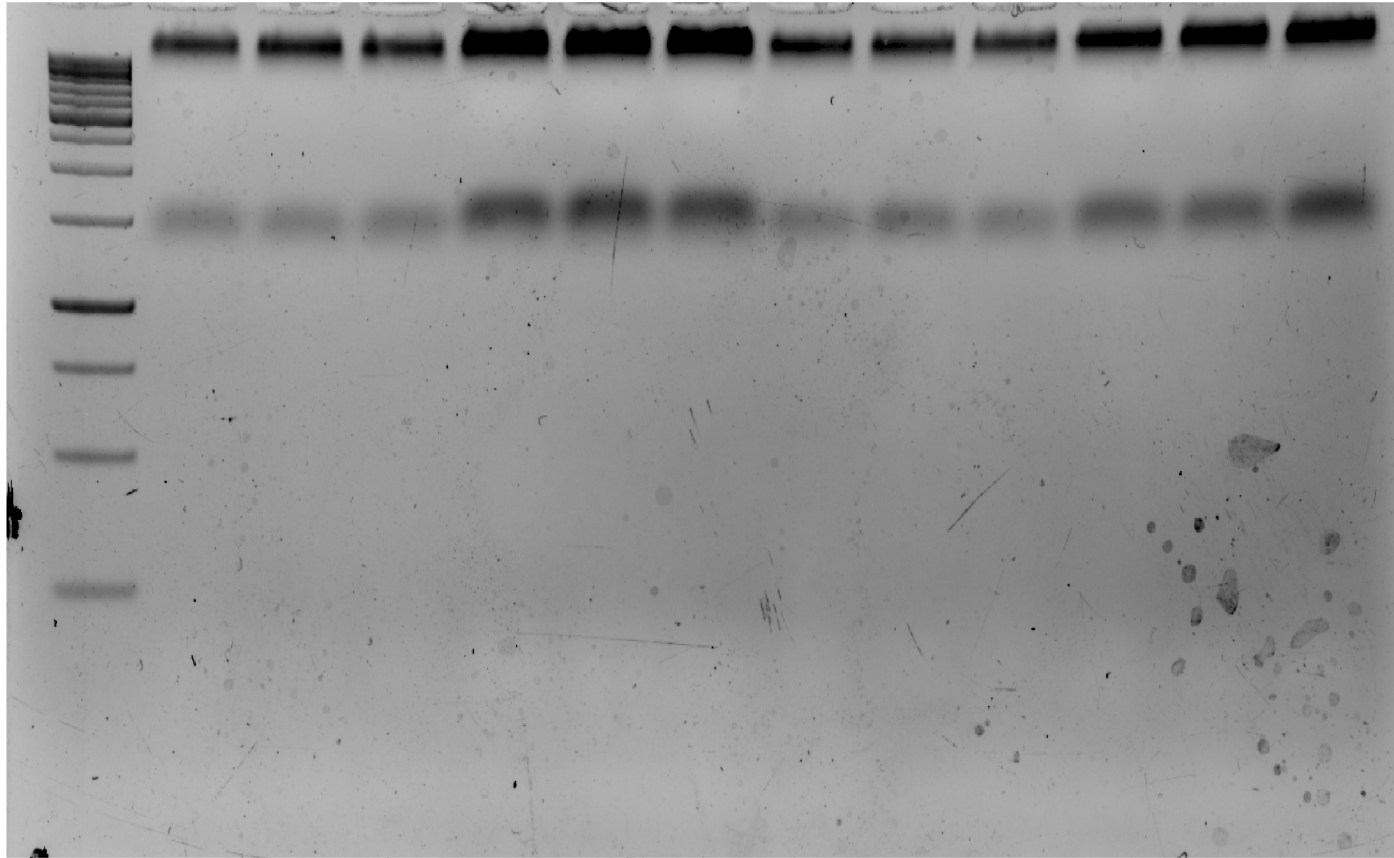

# P38 MAPK

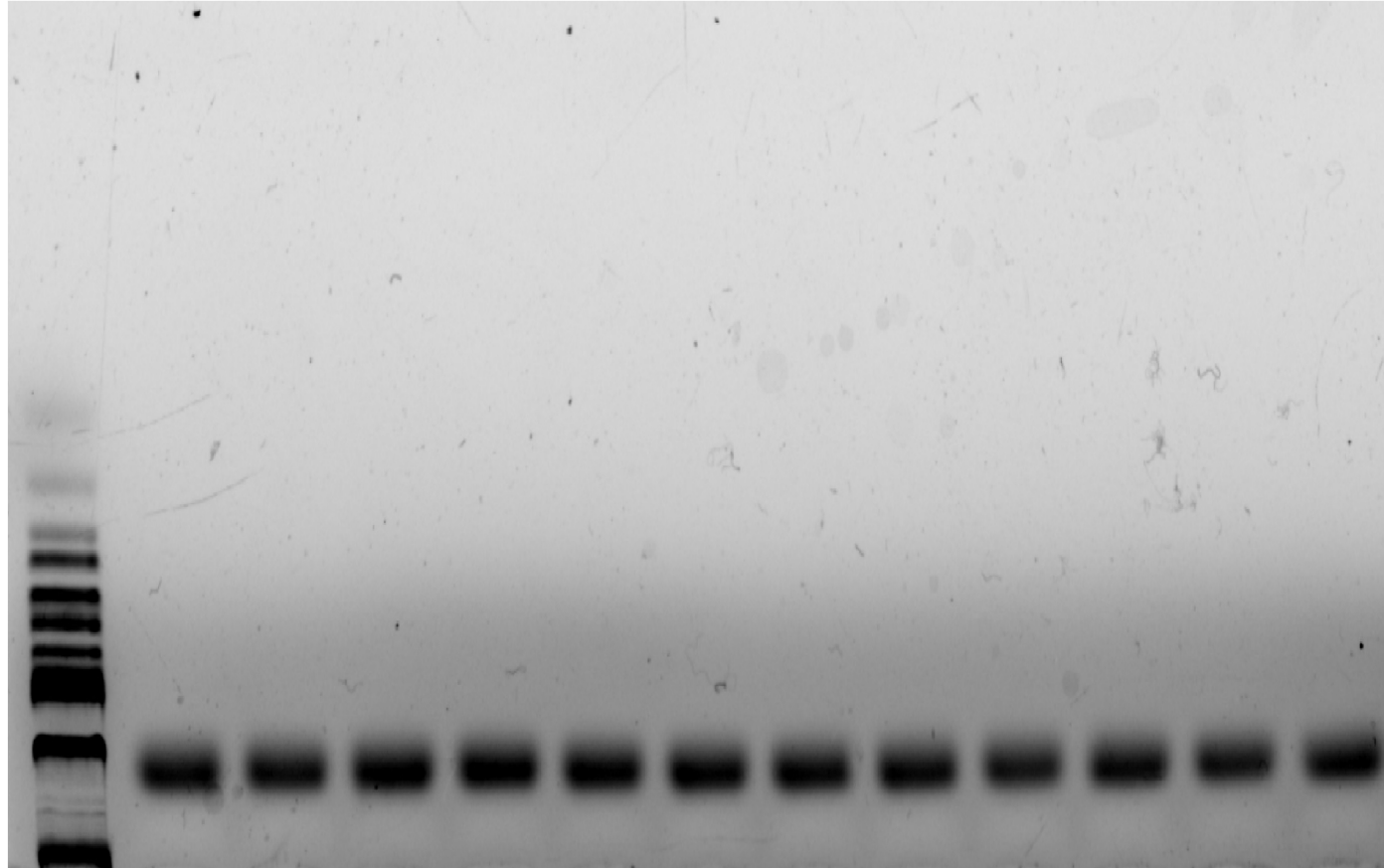

p-p38 MAPK

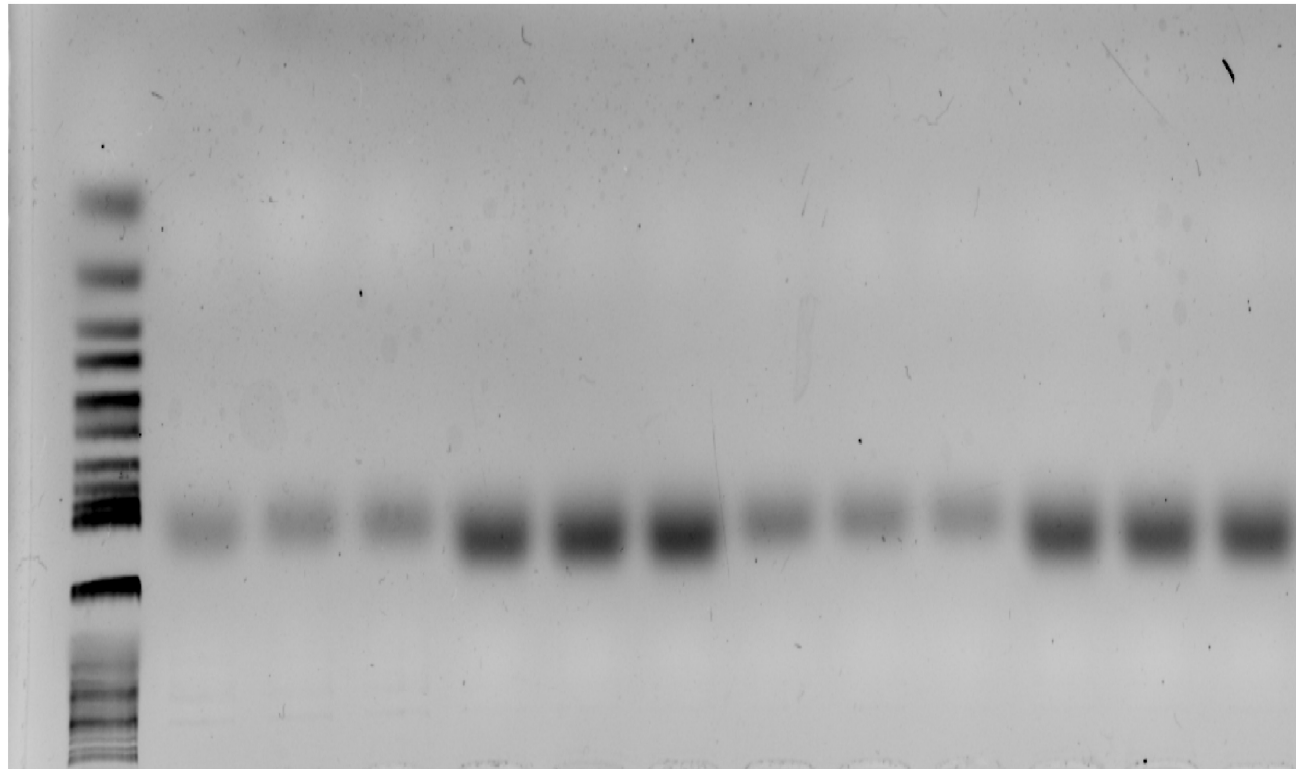

ERK1/2

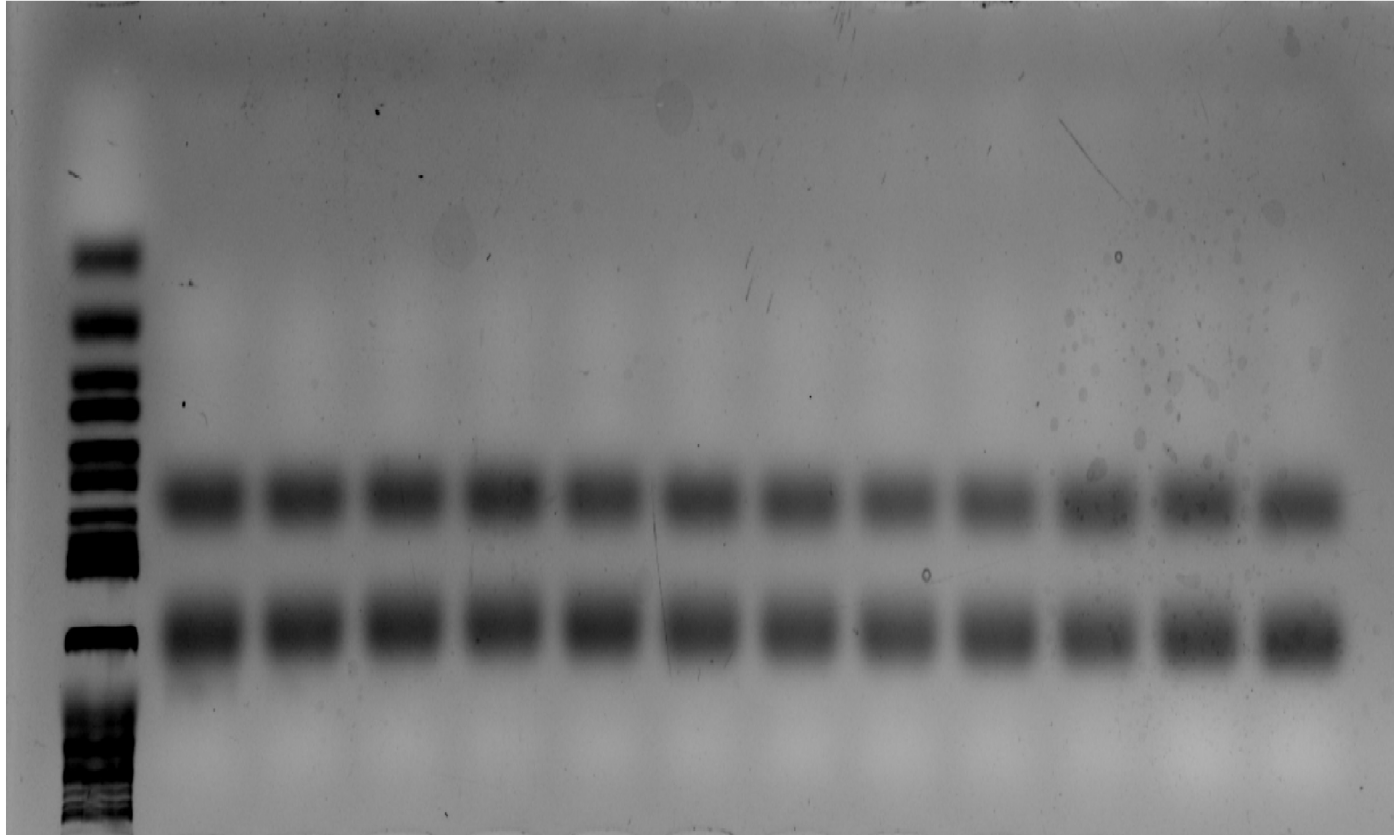

P-ERK1/2

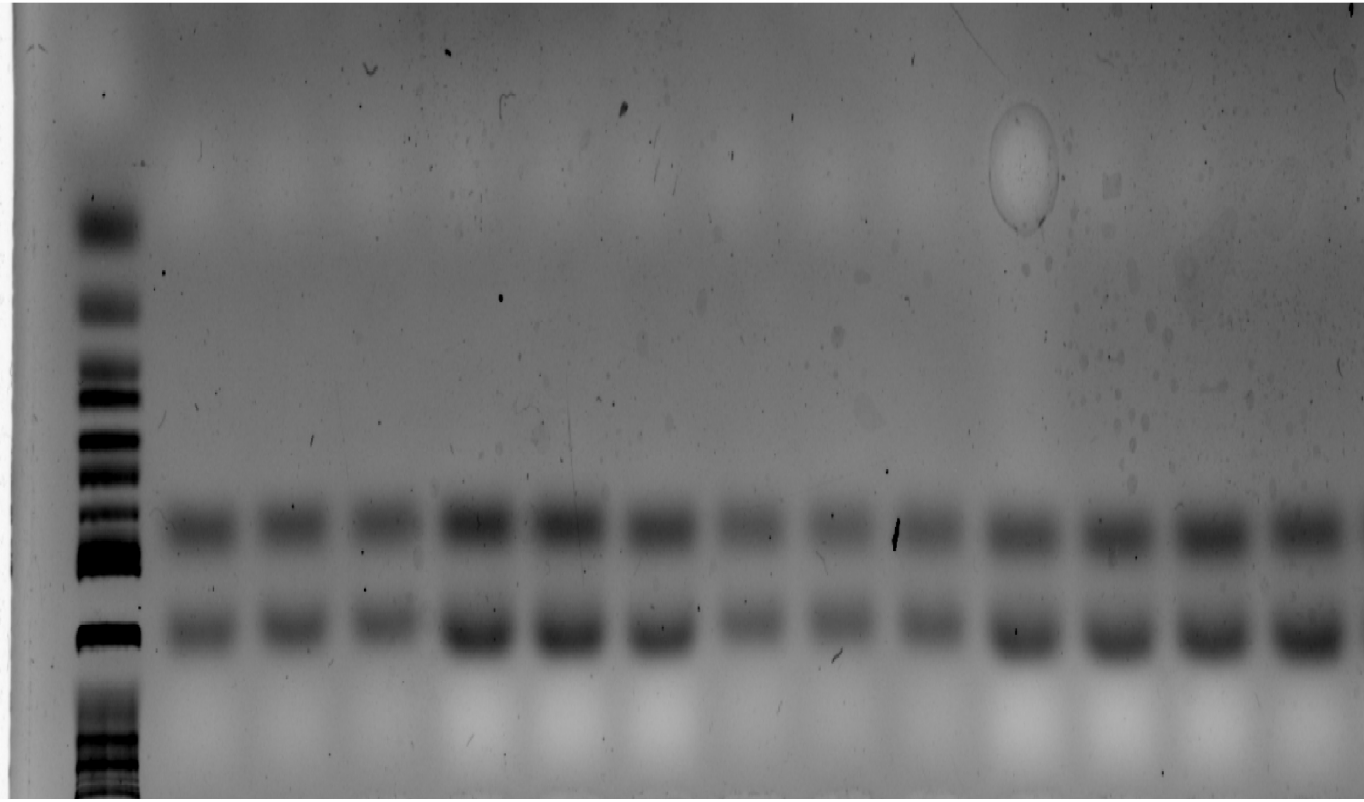

B-actin

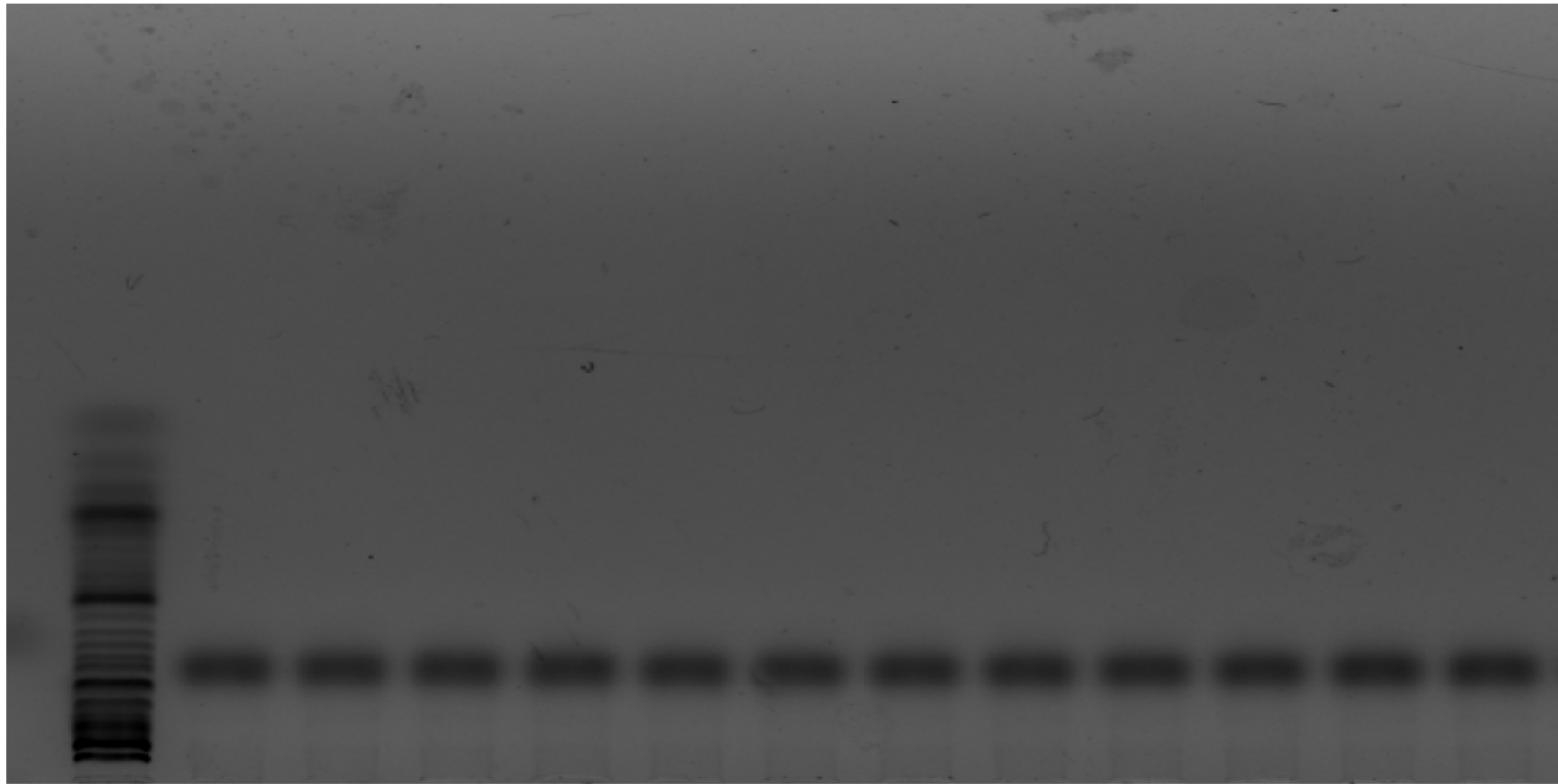

Supplement: Supplementary file 2 — Supplementary file2 (PDF 8055 kb) [file 12272_2022_1391_MOESM2_ESM.pdf]
